# Impact of an eight-week isocaloric vegan dietary intervention on hemogram parameters and lymphocyte subsets: a randomized-controlled trial

**DOI:** 10.1186/s12916-025-04612-y

**Published:** 2026-01-10

**Authors:** Julian Herter, Frieda Stübing, Volker Lüth, Ann-Kathrin Lederer, Ulrich Salzer, Ana Cecilia Venhoff, Bettina Sehnert, Luciana Hannibal, Reinhard Edmund Voll, Roman Huber, Maximilian Andreas Storz

**Affiliations:** 1 Department of Internal Medicine II, Centre for Complementary Medicine, Medical Center—University of Freiburg, Faculty of Medicine, University of Freiburg, Freiburg, 79106 Germany; 2 Department of General, Visceral and Transplant Surgery, University Medical Centre of the Johannes Gutenberg University, Langenbeckstraße 1, Mainz, 55131 Germany; 3 Department of Rheumatology and Clinical Immunology, Medical Center—University of Freiburg, Faculty of Medicine, University of Freiburg, Freiburg, Germany; 4 Laboratory of Clinical Biochemistry and Metabolism, Department of General Pediatrics, Adolescent Medicine and Neonatology, Faculty of Medicine, Medical Center, University of Freiburg, Freiburg, 79106 Germany; 5 Translational Metabolomics Core Facility, Faculty of Medicine, Medical Center, University of Freiburg, Freiburg, 79106 Germany; 6 CIBSS—Centre for Integrative Biological Signalling Studies, University of Freiburg, Freiburg, 79106 Germany; 7 Center of Chronic Immunodeficiency (CCI), Medical Center—University of Freiburg, Faculty of Medicine, University of Freiburg, Freiburg, 79106 Germany

**Keywords:** Clinical trial, Diet, Vegan, Nutrition, Immune system, Immunophenotyping, Inflammation, Cell proliferation

## Abstract

**Background:**

Whole food plant-based diets exert anti-inflammatory properties and have been associated with clinical improvements in patients with autoimmune disorders. The underlying mechanisms remain poorly understood and functional insights into nutrient-host physiology cross-talks are urgently warranted. The present study investigated the effects of an isocaloric 8-week vegan diet (VD) intervention on whole blood count parameters and lymphoid composition in comparison to a meat-rich diet (MD).

**Methods:**

We conducted a two-arm, monocentric randomized-controlled trial with healthy adults who were randomly allocated to either a MD or a VD for 8 consecutive weeks. Foods of animal origin were not permitted on the VD, whereas participants in the MD group were asked to consume at least 150 g of meat per day.

**Results:**

Fifty-seven participants completed the study. At week 8, significant between-group differences were found for the white blood cell count (median (interquartile range): 5.17 (1.62) *10^3^/µL in the VD group vs. 5.39 (1.92) *10^3^/µL in the MD group, *p* = 0.029) and the lymphocyte count (1.80 ± 0.53 *10^3^/µL in the VD group vs. 2.06 (0.74) *10^3^/µL in the MD group, *p* = 0.049). This difference was driven by an increase in lymphocytes in MD group participants over the course of the study. Median change scores in platelets differed between VD and MD participants (− 21 (− 31) *10^3^/µL in the VD group vs. − 1.21 ± 28.37 *10^3^/µL in the MD group, *p* = 0.035) and so did the neutrophil change scores (− 0.17 (− 0.31) *10^3^/µL vs. 0.13 (0.50) *10^3^/µL, *p* = 0.034). Mixed models for repeated measures with a time-diet interaction as a fixed effect suggested that changes in white blood cells were driven by the diet factor alone (contrast: − 0.50 (95% CI: − 0.99–(− 0.01)), *p* = 0.046). Immunophenotyping results suggested significant between-group differences in CD3^+^ and CD8^+^ T-cells, and CD19^+^ B-cells after 8 weeks. CD19^+^ B-cells decreased significantly in the vegan group (214.77 ± 96.64 at baseline vs. 171.56 (102.73) cells/µL at week 8).

**Conclusions:**

The present study suggests that a VD, in comparison to a MD, reduces the number of various immune cells even in healthy individuals. A VD may thus exert anti-inflammatory properties.

**Trial registration:**

Registered at Deutsches Register Klinischer Studien: DRKS00031541.

**Supplementary Information:**

The online version contains supplementary material available at 10.1186/s12916-025-04612-y.

## Background

Nutrition strongly affects numerous physiological processes, including those related to the immune system [1–3]. Pathways that link nutrition with host immunity represent an important opportunity to develop effective therapeutic dietary interventions and personalized nutritional approaches in the context of various diseases [1, 4]. A prominent example is rheumatoid arthritis (RA), a debilitating chronic inflammatory autoimmune disease, causing joint inflammation, pain, and potentially irreversible damage [5].

Several clinical studies demonstrated that a whole-food plant-based vegan diet (VD) may lead to noticeable clinical improvements in patients with RA [5–10]. Said improvements were frequently attributed to an altered ratio of n-6 to n-3 fatty acids, an increased intake of anti-inflammatory antioxidants, and—above all—weight loss [10, 11]. However, it is of paramount importance to recognize that the effects of a VD in patients with RA may not be solely attributed to weight reduction [10]. Sköldstam et al. suggested that weight reduction strategies in RA patients have a smaller influence on RA inflammation than commonly anticipated, particularly in the context of vegetarian and vegan diets [12].

Diet composition itself appears to be a critical determinant of a particular diet’s anti-inflammatory potential [13]. A diet’s macronutrient composition, fiber and iron content may be particularly important in modulating the inflammatory response. While diets abundant in animal foods may directly trigger pro-inflammatory cellular states [14, 15], whole-food plant-based diets exert anti-inflammatory properties possibly by providing a higher intake of polyphenols, flavonoids, and anti-inflammatory polyunsaturated fatty acids [3].

How nutrition exactly impacts human immunity remains largely unknown [1]. Functional insights into nutrient-host physiology cross-talks are urgently warranted to allow for targeted dietary interventions [1]. Our research group conducted several studies in this context, which aimed at a better understanding of the factors that mediate potentially beneficial effects of vegan diets. In a series of epidemiological and clinical studies, we examined dietary components and pathways other than weight loss, which may explain the anti-inflammatory characteristics of vegan diets [16]. In 2017, we conducted a clinical trial with *n* = 53 healthy omnivores who were randomly allocated to either an isocaloric vegan or meat-rich diet (MD) for 4 weeks. Controlling for weight loss, the vegan intervention resulted in a significant reduction in neutrophilic granulocytes, monocytes, and platelets [16].

The herein presented study constitutes an extended repetition study with different participants but a comparable design, a longer study duration of 8 weeks, an improved and intensified dietary counseling strategy, and a more comprehensive confounder and mediator assessment. The primary aim was to investigate the effects of an isocaloric VD on whole blood count parameters, lymphoid composition, and other systemic inflammatory markers after an 8-week dietary intervention in comparison to a MD.

## Methods

### Study design and general information

**Fig. 1:**
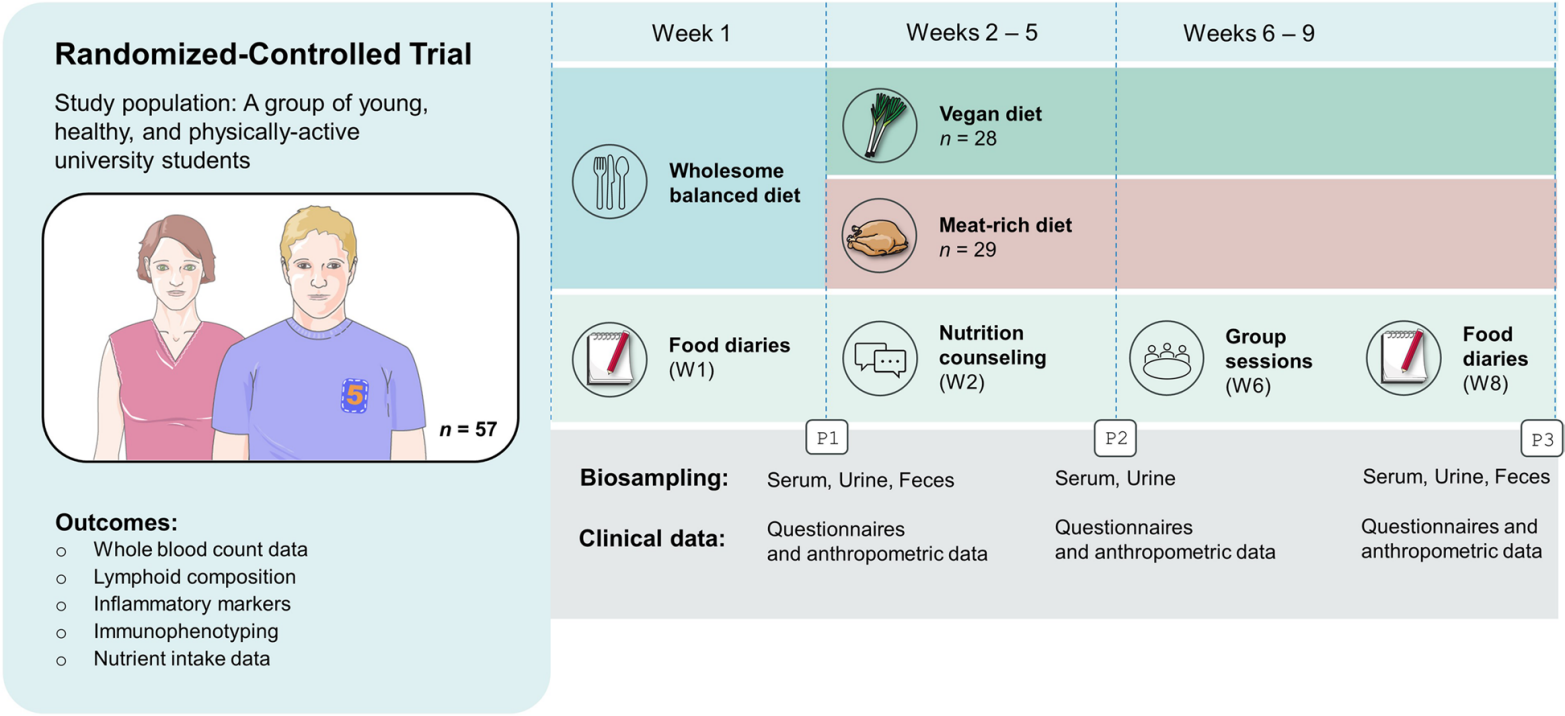
Course of the study: a schematic overview

The study design has been described in detail elsewhere [17]. In brief, we conducted a two-arm, monocentric randomized-controlled trial with young and healthy omnivorous individuals willing to modify their diet for a period of 8 weeks. The study was initiated by the Department of Internal Medicine II—Center for Complimentary Medicine at the University Medical Center of Freiburg. A total of *n* = 67 participants were randomly allocated to either a MD or a VD for eight consecutive weeks. Foods of animal origin were not permitted on the VD, whereas participants in the MD group were asked to consume at least 150 g of meat per day. No restrictions were imposed regarding the type of meat, although fish and fish products were not considered to fulfill the study criteria. The main difference between the two diets was the presence or absence of animal products. The degree of food processing was not explicitly controlled in our study. Participants in the VD group were encouraged to emphasize whole foods; this focus was not explicitly set in the MD group [17]. No meals were provided at any stage of the study. Instead, participants were free to select the foods they liked as long as their choices were in accordance with the study protocol. Figure 1 displays a study scheme, highlighting key aspects of this dietary intervention study.

This multi-topic study was performed between April and June 2023 with the intention to enhance our understanding of a vegan diet’s impact on immune system-related outcomes, such as lymphoid composition and immune signatures. Details regarding the recruitment process and regarding the eligibility criteria have been shared earlier [17]. The latter were selected to ensure a young and healthy population, with a limited number of non-dietary confounders. As such, we did not consider individuals with chronic health conditions or a regular medication. Pregnancy, lactation, and eating disorders were additional exclusion criteria. Special attention was paid to the participants’ past medical history: those who reported an infection within 14 days prior to the study were not considered for recruitment. A full list of the in- and exclusion criteria may be found in Herter et al. [17]. The study covered a wide range of topics related to the dietary intervention, including dietary and supplementation data, laboratory data as well as anthropometric and physical activity data [17].

### Primary outcome

The major study aim was to investigate the effects of an 8-week vegan dietary intervention on immune system-related outcomes. The between-group difference in mean/median change scores (baseline vs. week 8) in the number of neutrophil granulocytes was selected as the primary outcome. The sample size calculation was performed based on this outcome and in light of a previous study conducted by our group, suggesting a strong effect size (Cohen’s *d* = 1.0) for the changes in neutrophilic granulocytes subsequent to a 4-week dietary intervention [16]. Taking this into account and considering an anticipated statistical power of 95%, we calculated that *n* = 54 participants would be required to detect a statistical difference of *p* < 0.05 between both groups.

### Secondary outcome

As for the secondary outcomes of the study, we selected between-group differences in mean/median change scores in the number of lymphocytes and monocytes after the 8-week dietary intervention.

### Nutrient intake data assessment and nutritional coaching

The methods for the assessment of nutrient intake data have been described earlier in detail [17]. Dietary intake was estimated at baseline and during the last week of the study. Intake assessment was based on a 3-day weighed food diary following established procedures [17–19]. For the analysis of the food diaries, we used NutriGuide® diet software (version 4.9, Nutri-Science GmbH, Jacobistr. 39, 79104 Freiburg im Breisgau, Germany). The study included multiple group seminars, nutritional coaching sessions, and nutrient-specific weekly newsletters to increase the participants’ dietary adherence [17]. A study-specific telephone hotline was installed to allow for quick and regular contact between the study personnel and the study participants related to all dietary inquiries.

### Subpopulation-specific inclusion and exclusion criteria

The general in- and exclusion criteria have been presented earlier in detail [17]. A total of *n* = 65 participants completed the study. For the immune system-related analysis, however, not all participants could be considered. Participants who reported an infection 14 days prior to a scheduled venipuncture were not considered for this particular analysis. Likewise, those who denied an infection but showed elevated leukocyte counts > 9800/μL or elevated high-sensitive C-reactive protein (hs-CRP) levels (≥ 5 mg/l) were excluded, as well. We deemed this important to avoid any false attributions so that changes in inflammatory markers were not attributed to a respective diet in the context of a known prior infection. Likewise, we excluded participants with an hs-CRP-difference ≥ 3 mg/l from baseline to week 8, which may not be explained by a short-term dietary intervention alone [20, 21].

### Biomaterial acquisition and processing

Blood samples were taken early in the morning hours between 7 and 9:30 am after a fasting period of at least 10 h [17]. The analysis of the whole blood count and other clinical chemistry parameters (including a lipid panel, albumin, creatinine, CRP, HbA1c, an iron panel, and vitamin B12) was performed immediately by the accredited Central Laboratory of the University Medical Center of Freiburg (Institute of Clinical Chemistry and Laboratory Medicine, Medical Centre—University of Freiburg, Hugstetter Str. 55, 79106 Freiburg im Breisgau, Germany). Holotranscobalamin was measured externally at the accredited laboratory MVZ Clotten Labor, Dr. Haas, Dr. Raif & Kollegen GbR (Merzhauser Str. 112, 79100 Freiburg im Breisgau, Germany). For technical reasons, folate, zinc, and copper were measured 3 months later from frozen materials (see below). The whole blood samples for the lymphocyte subpopulation phenotyping and the extraction of peripheral blood mononuclear cells were stored in the dark at room temperature and processed within 3 h after venipuncture. All laboratory analyses were performed blinded for the dietary group assignment.

### Lymphocyte subpopulation phenotyping

Phenotyping of T-, B-, and NK cells within the lymphocyte population was performed by a whole blood staining lyse-no wash protocol (Optilyse B, Beckman Coulter) using six color flow cytometry with the following fluorochrome-conjugated antibodies: BV421 anti-CD3 (clone UCHT1; Biolegend), APC anti-CD4 (clone SK3; Becton Dickinson), FITC anti-CD8 (clone B9.11; Beckman Coulter Immunotech), PE anti-CD16 (clone 3G8; Beckman Coulter Immunotech), PE-Cy7 anti-CD19 (clone J3-119; Beckman Coulter Immunotech), PerCP anti-CD45 (clone HI30; Biolegend), PE anti-CD56 (clone N901; Beckman Coulter Immunotech). Fixed antibody labeled cells were analyzed within 24 h by flow cytometry (Navios; Beckman Coulter). Absolute cell counts were calculated using a two-platform method with leucocyte and lymphocyte counts determined by a hemocytometer. Flow cytometric data analysis was performed with the help of Kaluza Software 2.1 (Beckman Coulter).

### Amino acid and fatty acid profiling

Measurement of the amino acids was performed by the Translational Metabolomic Core Facility, Department for Pediatrics, University Hospital of Freiburg. Amino acids were determined as previously published [16]. A commercially available standardized amino acid mixture was utilized to generate a calibration curve for amino acids and urea cycle intermediates (Amino acid standards, physiological, Sigma, Nr. A9906-10ML). Calibration curves for all other metabolites were prepared from individual stock solutions prepared in house. The concentrations of metabolites in urine were normalized by the concentration of creatinine. Quantitation accuracy was examined by monitoring homocysteine and methylmalonic acid concentrations in an external quality control, namely, the Control Special Assays in Serum, European Research Network for the evaluation and improvement of screening, diagnosis, and treatment of Inherited disorders of Metabolism (ERNDIM) IQCS, SAS-02.1 and SAS-02.2 from MCA Laboratories, Winterswijk, Netherlands. For all other metabolites, quantitation trueness was tested by examining metabolite concentrations in plasma and urine samples from a previously validated sample isolated from a healthy control individual with respect to standard reference ranges and metabolite measurements performed independently in a diagnostic laboratory, using the same calibration curves and LC–MS/MS running conditions. Quantification of metabolites was carried out with Analyst® 1.7.2 software, 2022 AB Sciex. Fatty acid profiling was carried out by the Laboratory of Clinical Biochemistry and Metabolism, Department for Pediatrics, University Hospital of Freiburg, as described in prior work [16, 22].

### Covariates

Selected covariates with a known effect on the immune system were considered for this study. For this particular analysis, we registered physical activity levels (using the International Physical Activity Questionnaire—Short Form (IPAQ-SF) [23]), dietary supplementation behavior as well as subjective social status (as assessed by the German version of the MacArthur Scale of Subjective Social Status (MASSS) [24]). Participants were asked *not* to take any supplements during the intervention. Nevertheless, we inquired about dietary supplement intake at week 8.

### Statistical analysis

The statistical analysis was performed by MAS and JH using Stata 14 (StataCorp. 2015. Stata Statistical Software: Release 14. College Station, TX: StataCorp LP). Stata’s Shapiro–Wilk test was used in conjunction with histograms to test for normality. Normally distributed variables were described with their mean and standard deviation, whereas not normally distributed data was presented with their median and corresponding interquartile range. Categorical variables were described as follows: number of observations (percentage).

Depending on the data distribution, parametric (paired and unpaired two-tailed *t*-tests) and non-parametric tests (Wilcoxon rank sum test, Mann–Whitney *U* test) were used to test for statistically significant within-group (*pre*- vs. *post*-intervention) and between-group differences (*baseline* vs. *end of the study*) in continuous variables. We used the chi-squared test, Fisher’s exact test, and McNemar test where applicable for the comparison of the proportions of categorical variables. Scatterplots, Pearson’s product moment correlation coefficients, and Spearman’s correlation coefficients were calculated to examine potential associations between nutrient intake data and selected hemogram parameters (e.g., the platelet, neutrophil, lymphocyte, white blood cell, and monocyte count). Finally, repeated measures analysis of variance (rm-ANOVA) was performed for selected outcomes of interest to test for the between-subject factor of diet (two levels: meat-rich diet vs. vegan diet) and the within-subject factor of time (three levels: week 0, week 4, and week 8). Mauchly’s test of sphericity was used to test whether or not the assumption of sphericity was met in the rm-ANOVAs. The pooled within-subject covariance matrix was inspected carefully to assess whether compound symmetry was given.

We also ran MMRM (mixed model repeated measures), which was deemed a suitable approach for repeated measures data (blood count outcomes). Said models are a popular approach in the context of randomized clinical trials, which repeatedly measure individuals over time (in our case three measurements) [25]. Following an approach by Bartlett, we specified no participant level random effect [25]. Instead, our model modeled the correlation with the repeated measures over time by specifying that the residual errors were correlated. This was done to reduce the odds of model misspecification. In our case, residual errors were assumed to be from an unstructured covariance matrix. As for the “fixed effects,” we specified the time-diet interaction. The model was fitted using REML (restricted maximum likelihood) and modifications by Kenward and Roger as explained elsewhere [25, 26]. Stata’s contrast command with the diet-time interaction term was then used for a joint test of the interaction including main effects. Interactions were subsequently graphed with the margins function and marginsplots command. Statistical significance was determined at *α* = 0.05.

Due to the high number of statistical tests performed, we deemed it important to control the family-wise error rate when performing multiple hypothesis tests. As done earlier [17], we used Stata’s user-written command “wyoung,” calculating adjusted *p* values using the free step-down resampling methodology of Westfall and Young [27]. A precursor to the Romano-Wolf procedure [17, 27], this command allows for dependence amongst *p* values. The Westfall-Young correction was performed using 10,000 bootstraps. Familywise-error rate-adjusted Westfall-Young *p* values may be obtained from the legend of the respective tables. This procedure was not performed for correlation analyses.

### Ethical considerations

In January 2023, the local ethics committee approved this study before onset (approval number: 22–1474-S1). Written consent was obtained from all study participants. The study was registered prospectively before its onset (DRKS00031541) [28].

## Results

**Fig. 2:**
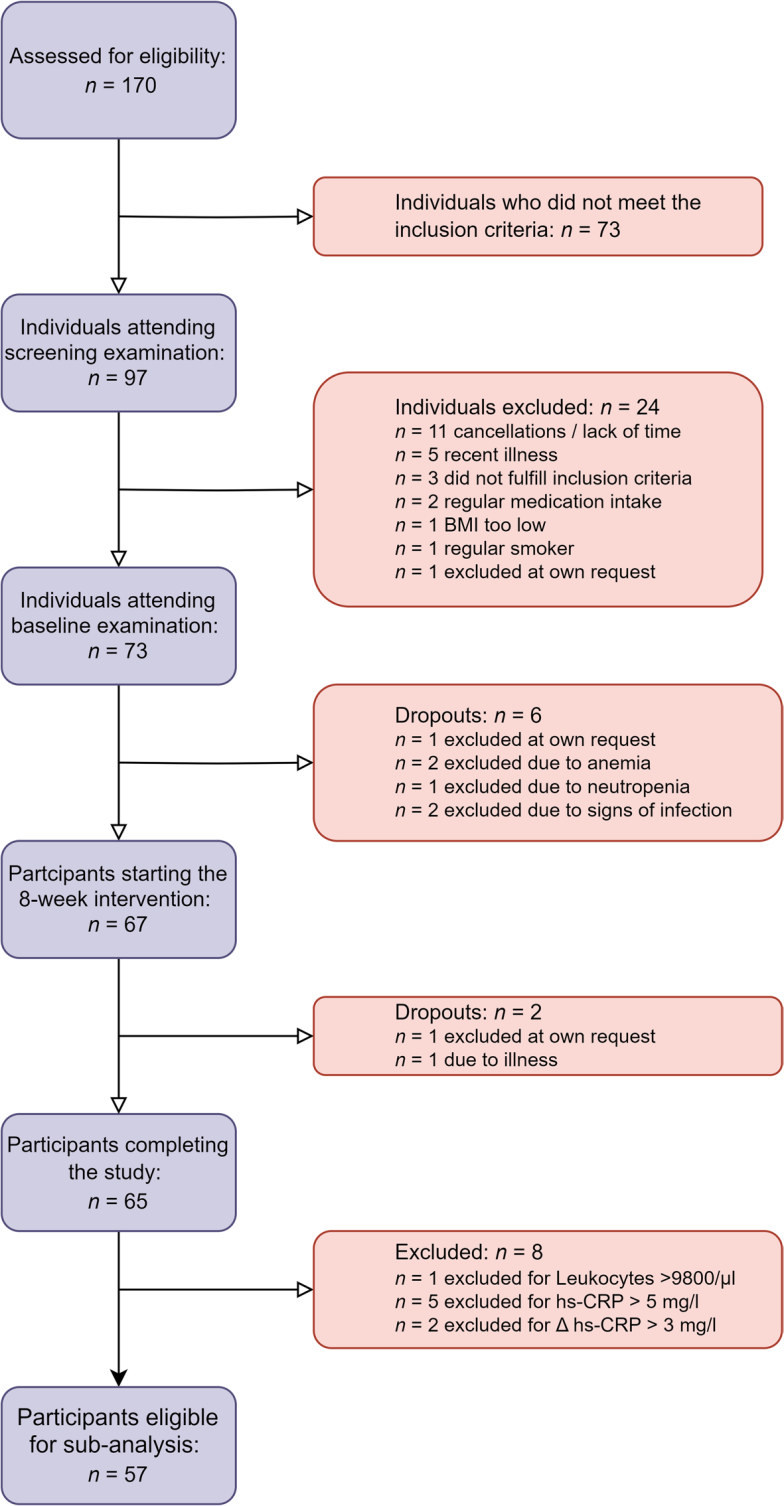
Participant inclusion flowchart

Figure 2 displays a participant inclusion flowchart, providing an explanation for inclusion and exclusion of participants in our study. A total of *n* = 57 participants were included in the final analysis.

### Sample characteristics

Table 1 shows the sample characteristics of the participants included in this sub-analysis. Males comprised almost 2/3 of the study sample (*n* = 35, 61.40%), whereas females accounted for approximately 39% (*n* = 22, 38.60%). Approximately 60% of the sample were highly active in terms of physical activity (health-enhancing physical activity, HEPA). As reported earlier, the sample was mainly composed of students, and all participants had at least finished secondary education with the German Abitur (the highest national high-school diploma) or a higher degree [17]. No significant baseline differences were observed for age, the body mass index, the physical activity level, and the educational level. Participants lost, on average, 1.32 kg of weight after 8 weeks in the study, although they were encouraged to maintain their body weight as constant as possible. There was no statistically significant between-group difference in the mean weight change score from week 8 to baseline (see Additional file 1: Table S1).

**Table 1:** Sample characteristics including sociodemographic, anthropometric, and physical activity data at baseline, after 4 weeks, and after 8 weeks of the dietary intervention

Variable	Complete sample (*n* = 57)	Vegan group (*n* = 28)	Meat-rich group (*n* = 29)	*p* value
Baseline				
Sex				0.916^c^
Male	*n* = 35 (61.40%)	*n* = 17 (60.71%)	*n* = 18 (62.07%)	
Female	*n* = 22 (38.60%)	*n* = 11 (39.39%)	*n* = 11 (37.93%)	
Age (years)	23 (3)	22 (2)	24 (3)	0.251^d^
Weight (kg)	74.45 ± 10^a^	74.58 ± 9.73^b^	73.33 ± 10.41	0.927^e^
Height (cm)	176.88 ± 9.04^a^	177.94 ± 8.79^b^	178.00 (17)	0.566^d^
BMI (kg/m^2^)	23.36 (3.14)^a^	22.92 (2.87)^b^	23.94 ± 2.12	0.417^d^
MASSS	6.89 ± 1.11	7.00 ± 1.15	6.79 ± 1.08	0.488^e^
Physical activity level				0.291^f^
Inactive	*n* = 2 (3.51%)	*n* = 2 (7.14%)	*n* = 0 (0%)	
Minimally active	*n* = 22 (38.60%)	*n* = 12 (42.86%)	*n* = 10 (34.48%)	
HEPA active	*n* = 33 (57.89%)	*n* = 14 (50.00%)	*n* = 19 (65.52%)	
Highest education level				0.218^c^
German “Abitur”	*n* = 45 (78.95%)	*n* = 24 (85.71%)	*n* = 21 (72.41%)	
University degree	*n* = 12 (21.05%)	*n* = 4 (14.29%)	*n* = 8 (27.59%)	
Week 4				
Weight (kg)	74.05 ± 9.90	74.33 ± 9.54	73.79 ± 10.4	0.841^e^
Height (cm)	177.19 ± 8.82	178.27 ± 8.51	178.00 (17.5)	0.615^d^
BMI (kg/m^2^)	23.31 (2.67)**	23.06 (2.85)**	23.69 ± 2.08*	0.555^d^
Physical activity level				1^f^
Inactive	*n* = 1 (1.75%)	*n* = 0 (0%)	*n* = 1 (3.45%)	
Minimally active	*n* = 23 (40.35%)	*n* = 12 (42.86%)	*n* = 11 (37.93%)	
HEPA active	*n* = 33 (57.89%)	*n* = 16 (57.14%)	*n* = 17 (58.62%)	
Week 8				
Weight (kg)	73.28 ± 10.12	73.39 ± 9.99	73.18 ± 10.41	0.937^e^
Height (cm)	177.40 ± 8.84	178.55 ± 8.56	177.50 (17)	0.510^d^
BMI (kg/m^2^)**	22.92 (2.96)	22.95 ± 1.96**	23.46 ± 2.19**	0.356^e^
MASSS	7.00 ± 1.36	7.07 ± 1.44	7.00 (2)	0.824^d^
Physical activity level				0.395^f^
Inactive	*n* = 1 (1.75%)	*n* = 1 (3.57%)	*n* = 0 (0%)	
Minimally active	*n* = 18 (31.58%)	*n* = 7 (25.00%)	*n* = 11 (37.93%)	
HEPA active	*n* = 38 (66.67%)	*n* = 20 (71.43%)	*n* = 18 (62.07%)	

Continuous data displayed as mean ± SD if normally distributed or as median (IQR) if not normally distributed. Categorical data displayed as *n* = *x* (%). Significant *p* values shown in bold. * indicates significant differences in comparison to baseline at a *p* value < 0.05; ** indicates significant differences in comparison to baseline at a *p* value < 0.001

*MASSS *MacArthur Scale of Subjective Social Status, *HEPA *health-enhancing physical activity

^a^Based on 56 observations

^b^Based on *n* = 27 observations (no anthropometric data available for one person at baseline)

^c^Based on chi-square analyses

^d^Based on Wilcoxon (Mann–Whitney) rank sum test analyses

^e^Based on Student’s *t*-test analyses

^f^Based on Fisher’s exact test. Columns may not equal 100% due to rounding

### Laboratory data: complete blood count

Table 2 shows the full blood count at baseline and after 4 and 8 weeks of the dietary intervention, respectively. The two groups did not differ statistically in any parameter at baseline. After 8 weeks, no significant between-group differences were observed for the neutrophil count (2.55 ± 0.63 * 1000/µL in the VD group vs. 2.93 ± 0.07 * 1000/µL in the MD group, *p* = 0.052). Significant intergroup differences were observed for the white blood cell count (5.39 (1.92) * 1000/µL in the MD group vs. 5.17 (1.61) * 1000/µL in the vegan group), the eosinophil count, and the lymphocyte count (2.06 (0.74) * 1000/µL vs. 1.80 ± 0.53 * 1000/µL). The difference in the white blood cell count remained significant after adjustment (Westfall-Young *p* value: 0.044), whereas this was not the case for the eosinophil count (Westfall-Young *p* value: 0.629). Additional file 1: Fig. S1 includes a strip plot visualizing the white blood cell count over the course of the study and depicts an increase in white blood cells in the MD group whereas no major changes were observed in the VD group. No adjustments for the primary (neutrophil count) and secondary outcomes (lymphocyte count) were performed. There was also a clear but non-significant trend at week 8 for the intergroup difference in the neutrophil count (lower absolute values in the VD group, higher absolute values in the MD group) (see Additional file 1: Fig. S2).

**Table 2:** Complete blood count data at baseline, after 4 weeks, and after 8 weeks of the dietary intervention

Laboratory value	Complete sample (*n* = 57)	Vegan group (*n* = 28)	Meat-rich group (*n* = 29)	*p* value
Baseline				
Red blood cells (million cells/µL)	4.74 ± 0.48	4.72 ± 0.47	4.77 ± 0.5	0.711^a^
Hemoglobin (g/dl)	14.25 ± 1.23	14.13 ± 1.16	14.37 ± 1.29	0.458^a^
Hematocrit (%)	42.27 ± 3.36	41.98 ± 3.24	42.56 ± 3.52	0.515^a^
Mean cell volume (fl)	89.38 ± 3.79	89.20 ± 3.81	89.54 ± 3.82	0.735^a^
Mean cell hemoglobin (pg)	30.13 ± 1.55	30.02 ± 1.58	30.23 ± 1.53	0.619^a^
Mean corpuscular hemoglobin concentration (g/dl)	33.72 ± 1.05	33.66 ± 0.96	33.77 ± 1.14	0.691^a^
Platelets (thousand/µL)	236.05 ± 52.91	233.29 ± 54.63	222.00 (89)	0.936^b^
White blood cells (thousand/µL)	5.14 (1.52)	5.35 ± 1.35	5.15 (1.39)	0.458^b^
Neutrophils (%)	51.50 ± 8.29	52.43 ± 7.73	50.60 ± 8.83	0.408^a^
Neutrophil counts (thousand/µL)	2.70 (0.93)	2.48 (1.14)	2.75 (0.86)	0.817^b^
Eosinophils (%)	2.30 (1.6)	2.00 (1.7)	2.50 (1.3)	0.180^b^
Eosinophil count (thousand/µL)	0.13 (0.09)	0.12 (0.1)	0.13 (0.06)	0.270^b^
Basophiles (%)	0.86 ± 0.31	0.81 ± 0.31	0.90 ± 0.31	0.306^a^
Basophile count (thousand/µL)	0.04 (0.02)	0.04 ± 0.02	0.05 ± 0.02	0.239^a^
Monocytes (%)	9.44 ± 2.00	9.05 ± 1.97	9.81 ± 1.99	0.153^a^
Monocyte count (thousand/µL)	0.51 ± 0.15	0.49 ± 0.18	0.53 ± 0.12	0.281^a^
Lymphocytes (%)	35.31 ± 7.14	34.94 ± 7.29	35.67 ± 7.11	0.706^a^
Lymphocyte count (thousand/µL)	1.82 (0.48)	1.83 ± 0.45	1.83 (0.56)	0.615^b^
Week 4				
Red blood cells (million cells/µL)	4.67 ± 0.43*	4.62 ± 0.37*	4.72 ± 0.48	0.389^a^
Hemoglobin (g/dl)	14.01 ± 1.17*	13.84 ± 0.95*	14.18 ± 1.34	0.285^a^
Hematocrit (%)	41.28 ± 2.98*	40.85 ± 2.48*	41.70 ± 3.38	0.283^a^
Mean cell volume (fl)	88.62 ± 3.48**	88.60 ± 3.34*	88.63 ± 3.67*	0.968^a^
Mean cell hemoglobin (pg)	30.06 ± 1.51	30.03 ± 1.53	30.10 ± 1.52	0.860^a^
Mean corpuscular hemoglobin concentration (g/dl)	33.92 ± 1.01*	33.88 ± 0.92*	33.96 ± 1.11	0.758^a^
Platelets (thousand/µL)	231.91 ± 52.24	220.21 ± 53.35*	243.21 ± 49.43	0.097^a^
White blood cells (thousand/µL)	5.41 ± 1.08	5.12 ± 1.08	5.7 ± 1.02	**0.040**^a^
Neutrophils (%)	50.03 ± 9.1	50.79 ± 10.19	49.3 ± 8.04	0.541^a^
Neutrophil counts (thousand/µL)	2.45 (1.06)	2.23 (1.17)	2.82 ± 0.76	0.185^b^
Eosinophils (%)	2.40 (1.6)	1.80 (1.70)	2.70 (1.30)	**0.036**^b^
Eosinophil count (thousand/µL)	0.13 (0.09)	0.10 (0.08)	0.15 (0.06)	**0.011**^b^
Basophiles (%)	0.80 (0.50)	0.82 ± 0.27	0.70 (0.50)	0.885^b^
Basophile count (thousand/µL)	0.04 (0.03)	0.40 ± 0.01	0.05 ± 0.02	0.127^a^
Monocytes (%)	9.91 ± 2.08	9.86 ± 2.32*	9.97 ± 1.86	0.851^a^
Monocyte count (thousand/µL)	0.54 ± 0.16	0.51 ± 0.16	0.56 (0.18)	0.116^b^
Lymphocytes (%)	36.04 ± 8.44	35.63 ± 9.11	36.44 ± 7.88	0.721^a^
Lymphocyte count (thousand/µL)	1.94 ± 0.55	1.80 ± 0.52	2.06 ± 0.55	0.070^a^
Week 8				
Red blood cells (million cells/µL)	4.62 ± 0.46*	4.57 ± 0.42*	4.67 ± 0.49	0.426^a^
Hemoglobin (g/dl)	13.86 ± 1.15**	13.71 ± 0.98*	14.01 ± 1.3*	0.332^a^
Hematocrit (%)	40.81 ± 3.18**	40.44 ± 2.79*	41.16 ± 3.52*	0.397^a^
Mean cell volume (fl)	88.5 ± 3.62*	88.67 ± 3.68	88.34 ± 3.61*	0.737^a^
Mean cell hemoglobin (pg)	30.07 ± 1.53	30.07 ± 1.47	30.08 ± 1.62	0.985^a^
Mean corpuscular hemoglobin concentration (g/dl)	33.98 ± 0.84*	33.92 ± 0.80	34.03 ± 0.88	0.605^a^
Platelets (thousand/µL)	228.54 ± 47.77	219.25 ± 49.79*	222.00 (73)	0.326^b^
White blood cells (thousand/µL)	5.33 (1.35)	5.43 ± 1.10	5.39 (1.92)	**0.029**^b^
Neutrophils (%)	50.42 ± 7.94	50.63 ± 7.90	50.21 ± 8.12	0.842^a^
Neutrophil counts (thousand/µL)	2.64 (0.98)	2.55 ± 0.63	2.93 ± 0.79	0.052^a^
Eosinophils (%)	2.40 (1.70)*	2.15 (1.55)	2.70 (1.60)	0.079^b^
Eosinophil count (thousand/µL)	0.12 (0.08)	0.12 (0.09)	0.16 (0.07)	**0.024**^b^
Basophiles (%)	0.89 ± 0.32	0.86 ± 0.30	0.92 ± 0.33	0.506^a^
Basophile count (thousand/µL)	0.05 (0.03)	0.04 ± 0.02	0.05 (0.04)	0.074^b^
Monocytes (%)	9.62 ± 2.18	8.85 (3.25)*	9.35 ± 1.67	0.873^b^
Monocyte count (thousand/µL)	0.52 ± 0.13	0.50 ± 0.14	0.53 (0.13)	0.237^b^
Lymphocytes (%)	35.77 ± 6.85	35.57 ± 6.97	35.97 ± 6.85	0.829^a^
Lymphocyte count (thousand/µL)	1.77 (0.73)	1.80 ± 0.53	2.06 (0.74)	**0.049**^b^

Continuous data displayed as mean ± SD if normally distributed or as median (IQR) if not normally distributed. Significant *p* values shown in bold. * indicates significant differences in comparison to baseline at a *p* value < 0.05; ** indicates significant differences in comparison to baseline at a *p* value < 0.001

The familywise error rate-adjusted Westfall-Young *p* values for differences in the white blood cell count and in the eosinophil count at week 8 (10 hypothesis tests, no adjustments for the primary and secondary outcomes were performed) were 0.044 and 0.629, respectively

^a^Based on Student’s *t*-test

^b^Based on Wilcoxon (Mann–Whitney) rank sum test

**Fig. 3:**
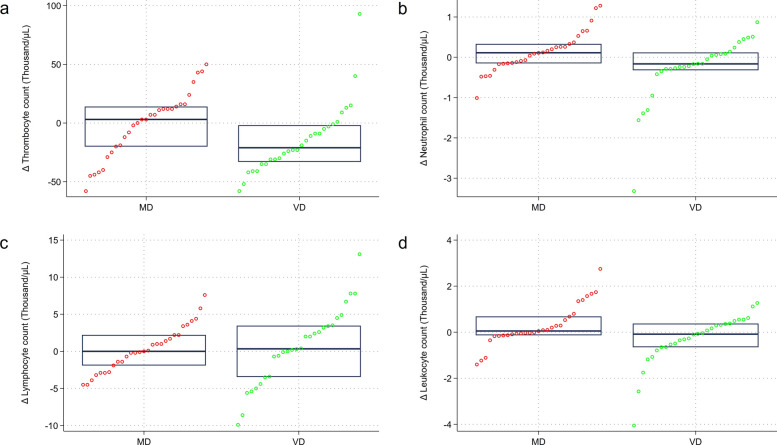
Median change scores in selected full blood count parameters by dietary group. The figure shows group-specific median change scores in the thrombocyte (**a**), neutrophil (**b**), lymphocyte (**c**), and white blood cell (leukocyte) count (**d**). Significant intergroup differences for the median change score were found for the thrombocyte count (*p* = 0.036) and the neutrophil count (*p* = 0.034)

In a subsequent step, we investigated mean/median change scores in the thrombocyte, neutrophil, lymphocyte, and white blood cell count. Figure 3 visualizes these differences in a group-specific matter. As shown in the top left (panel a), the median platelet counts significantly decreased in the VD group in comparison to the MD group (*p* = 0.036). Likewise, the median neutrophil count (panel b) also decreased significantly in the VD group when compared to the MD group (*p* = 0.034). The median change scores for the lymphocyte (c) and white blood cell count (d) did not differ significantly between the two groups.

### Laboratory data: clinical chemistry

Table 3 shows the available clinical chemistry parameters at baseline and after 8 weeks of the dietary intervention. The two groups did not differ in any parameter at baseline. No significant intergroup differences were observed regarding the iron panel after the dietary intervention. Lipid panel parameters, however, changed substantially, with a significant decrease in total cholesterol and LDL levels in the VD group. The non-supplemented dietary intervention also led to a significant and expected change in the vitamin B12 status of VD participants, with substantial decreases in holotranscobalamin and serum vitamin B12 levels. A minor but significant increase in serum folic acid levels was observed in the VD group. Median hs-CRP levels slightly increased in both groups, and the increase was significant in participants assigned to the vegan group, presumably due to their very low baseline values and a seasonal phenomenon (see Additional file 1: Fig. S3).

**Table 3:** Laboratory data at baseline and after 8 weeks of the dietary intervention: clinical chemistry

Laboratory value	Complete sample (*n* = 57)	Vegan group (*n* = 28)	Meat-rich group (*n* = 29)	*p* values
Baseline				
Albumin (g/dl)	4.82 ± 0.23	4.83 ± 0.19	4.81 ± 0.27	0.607^a^
Iron (μg/dl)	100.00 (67.00)	113.57 ± 44.58	84.00 (73.00)	0.202^b^
Ferritin (ng/ml)	44.00 (52.00)	39.00 (41.50)	51.00 (61.00)	0.260^b^
HbA1_C_ (%)	5.20 ± 0.25	5.19 ± 0.25	5.21 ± 0.25	0.834^a^
HbA1_C_ (mmol/mol)	33.25 ± 2.67	33.14 ± 2.69	33.36 ± 2.69	0.760^a^
High-density lipoprotein (mg/dl)	63.00 (17.00)	65.00 (17.50)	63.79 ± 15.40	0.755^b^
Low-density lipoprotein (mg/dl)	83.00 (30.00)	86.46 ± 20.51	85.00 (33.00)	0.811^b^
Cholesterol (mg/dl)	158.42 ± 28.15	156.21 ± 24.98	159.00 (34.00)	0.768^b^
High-sensitive CRP (mg/l)	0.40 (0.40)	0.35 (0.35)	0.50 (0.60)	0.161^b^
Serum transferrin receptor (mg/l)	2.70 (1.00)	2.68 ± 0.62	2.91 ± 0.83	0.241^a^
Serum triglycerides (mg/l)	64.00 (34.00)	63.00 (36.50)	65.00 (30.00)	0.987^b^
Holotranscobalamin (pmol/l)	64.90 (37.10)	67.20 ± 23.59	75.07 ± 27.94	0.256^a^
Transferrin (mg/dl)	283.82 ± 44.23	284.57 ± 46.93	283.10 ± 42.27	0.902^a^
Transferrin saturation (%)	27.00 (23.00)	30.50 (19.50)	20.00 (23.00)	0.307^b^
Uric acid (mg/dl)	4.84 ± 0.95	4.91 ± 1.01	4.76 ± 0.90	0.542^a^
Vitamin B12 (pg/ml)	342.00 (139.00)	342.50 (131.00)	324.00 (183.00)	0.582^b^
Copper (μg/dl)	87.00 (24.00)	86.00 (25.00)	90.00 (21.00)	0.384^b^
Zinc (μg/dl)	84.82 ± 9.94	84.75 ± 10.11	84.90 ± 9.95	0.956^a^
Folate (ng/ml)	8.50 (4.70)	8.95 (5.50)	8.00 (3.60)	0.533^b^
Week 8				
Albumin (g/dl)	4.74 ± 0.23*	4.74 ± 0.22*	4.74 ± 0.25	0.981^a^
Iron (μg/dl)	99.00 (67.00)	98.29 ± 40.77	105.00 ± 43.18	0.549^a^
Ferritin (ng/ml)	33.00 (39.00)	33.00 (37.50)	39.00 (40.00)	0.643^b^
HbA1_C_ (%)	5.23 ± 0.23	5.19 ± 0.24	5.27 ± 0.21	0.173^a^
HbA1_C_ (mmol/mol)	33.58 ± 2.44	33.14 ± 2.64	34.01 ± 2.19	0.178^a^
High-density lipoprotein (mg/dl)	58.00 (20.00)*	55.00 (17.50)*	63.00 (24.00)	0.411^b^
Low-density lipoprotein (mg/dl)	79.00 (24.00)**	72.96 ± 13.95**	86.31 ± 24.34	**0.014**^a^
Cholesterol (mg/dl)	142.00 (32.00)**	136.71 ± 22.14**	153.69 ± 32.04*	**0.024**^a^
High-sensitive CRP (mg/l)	0.60 (0.80)*	0.55 (1.00)*	0.70 (0.50)	0.791^b^
Serum transferrin receptor (mg/l)	2.78 ± 0.65	2.68 ± 0.61	2.88 ± 0.69	0.256^a^
Serum triglyceride (mg/l)	66.00 (30.00)	68.57 ± 19.20	67.00 (32.00)	0.678^b^
Holotranscobalamin (pmol/l)	67.06 ± 30.10	46.70 (34.60)**	80.90 ± 29.29	** < 0.001**^b^
Transferrin (mg/dl)	279.84 ± 42.45	280.25 ± 40.81	279.45 ± 44.69	0.944^a^
Transferrin saturation (%)	26.00 (18.00)	25.61 ± 11.90	27.59 ± 12.82	0.549^a^
Uric acid (mg/dl)	5.11 ± 0.91*	5.30 ± 0.97*	4.92 ± 0.83	0.115^a^
Vitamin B12 (pg/ml)	286.00 (150.00)**	269.00 (91.50)**	304.00 (228.00)	**0.021**^b^
Copper (μg/dl)	87.00 (24.00)	87.50 (19.50)	89.00 (16.00)	0.696^b^
Zinc (μg/dl)	83.60 ± 10.21	84.07 ± 10.93	83.14 ± 9.64	0.733^a^
Folate (ng/ml)	8.70 (5.40)	9.35 (4.90)*	8.00 (4.80)	**0.039**^b^

Continuous data displayed as mean ± SD if normally distributed or as median (IQR) if not normally distributed. * indicates significant differences in comparison to baseline at a *p* value < 0.05; ** indicates significant differences in comparison to baseline at a *p* value < 0.001

The familywise error rate-adjusted Westfall-Young p values for the intergroup differences in low-density lipoprotein, cholesterol, holotranscobalamin, vitamin B12,and folate at week 8 (16 hypothesis tests) were 0.154, 0.245, 0.002, 0.101, and 0.753, respectively

^a^Based on Student’s *t*-test

^b^Based on Wilcoxon (Mann–Whitney) rank sum test. Significant *p* values shown in bold

### Laboratory data: immunophenotyping

**Fig. 4:**
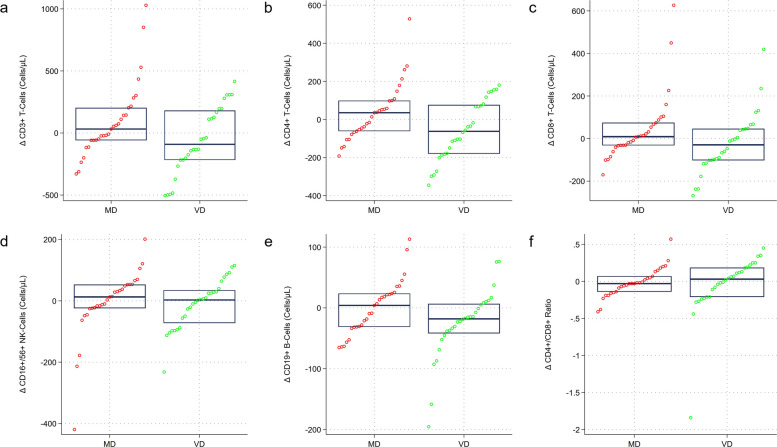
Median change scores in selected immunophenotyping parameters by dietary group. The figure shows group-specific median change scores in CD3^+^ T-cells (**a**), in CD4^+^ T-cells (**b**), in CD8^+^ T-cells (**c**), in CD16^+^/56^+^ NK-cells (**d**), in CD19^+^ B-cells (**e**), and in the CD4^+^/CD8^+^ ratio (**f**). No significant intergroup differences for the median change score were found, although trends were visible for CD3^+^ T-cells (**a**), CD4^+^ T-cells (**b**), in CD8^+^ T-cells (**c**), and CD19^+^ B-cells (**d**), with a decrease in all parameters in vegans

Table 4 shows the results of the immunophenotyping performed at baseline and after 8 weeks of the dietary intervention. Significant unadjusted intergroup differences in CD3^+^ T-cells, CD8^+^ T-cells, and CD19^+^ B-cells were found after 8 weeks of the dietary intervention. None of these differences remained significant when considering familywise error rate-adjusted *p* values (the Westfall-Young *p* values were 0.136, 0.193, and 0.409, respectively). CD19^+^ B-cells decreased significantly in the VD group. Figure 4 visualizes immunophenotyping differences in a group-specific matter, showing a decreasing but non-significant trend for CD3^+^ T-cells (panel a), CD4^+^ T-cells (panel b), CD8^+^ T-cells (panel c), and CD19^+^ B-cells (panel d) in vegans.

**Table 4:** Laboratory data at baseline and after 8 weeks of the dietary intervention: immunophenotyping

Laboratory value	Complete sample (*n* = 57)	Vegan group (*n* = 28)	Meat-rich group (*n* = 29)	*p* values
Baseline				
CD3^+^ T-cells (%)	75.44 ± 6.95	75.52 ± 7.08	75.36 ± 6.96	0.935^a^
CD4^+^ T-cells (%)	44.05 ± 6.42	45.37 ± 6.83	42.78 ± 5.82	0.130^a^
CD8^+^ T-cells (%)	26.41 ± 6.31	25.48 ± 6.04	26.61 (8.52)	0.389^b^
CD19^+^ B-cells (%)	11.76 ± 3.95	11.76 ± 4.23	11.76 ± 3.74	0.993^a^
CD16^+^/56^+^ NK-cells (%)	11.91 (7.22)	11.84 (5.23)	12.44 ± 5.34	0.723^b^
CD3^+^ T-cells (cells/μL)	1400.34 ± 347.99	1351.55 ± 306.07	1338.69 (367.57)	0.750^b^
CD4^+^ T-cells (cells/μL)	827.72 ± 219.50	823.49 ± 208.85	831.80 ± 232.95	0.888^b^
CD8^+^ T-cells (cells/μL)	478.48 (136.87)	465.21 ± 141.32	470.41 (160.76)	0.555^b^
CD19^+^ B-cells (cells/μL)	222.90 ± 95.60	214.77 ± 96.64	230.76 ± 95.61	0.533^a^
CD16^+^/56^+^ NK-cells (cells/μL)	214.21 (147.53)	211.15 (130.17)	224.61 (177.86)	0.434^b^
CD4^+^/CD8^+^ ratio	1.67 (0.60)	1.81 (0.73)	1.66 ± 0.47	0.225^b^
Week 8				
CD3^+^ T-cells (%)	76.05 ± 6.57	77.32 (7.52)	76.10 ± 6.59	0.621^b^
CD4^+^ T-cells (%)	44.28 ± 6.34	46.58 (11.41)	43.30 ± 6.09	0.238^b^
CD8^+^ T-cells (%)	26.86 ± 6.18	25.86 ± 5.85	27.29 (8.59)	0.334^b^
CD19^+^ B-cells (%)	11.14 ± 3.74*	10.82 ± 3.86*	11.45 ± 3.67	0.525^a^
CD16^+^/56^+^ NK-cells (%)	12.65 (5.81)	11.78 (4.43)	12.14 ± 5.00	0.823^b^
CD3^+^ T-cells (cells/μL)	1341.06 (526.65)	1294.97 ± 353.63	1561.80 (501.81)	**0.022**^b^
CD4^+^ T-cells (cells/μL)	820.36 ± 220.31	738.49 (304.59)	871.09 ± 214.72	0.062^b^
CD8^+^ T-cells (cells/μL)	468.70 (232.73)	443.16 ± 156.87	537.75 (194.15)	**0.017**^b^
CD19^+^ B-cells (cells/μL)	199.94 (127.86)	171.56 (102.73)*	231.43 ± 91.73	**0.046**^b^
CD16^+^/56^+^ NK-cells (cells/μL)	201.62 (133.22)	186.98 (88.79)	223.77 (171.65)	0.170^b^
CD4^+^/CD8^+^ ratio	1.75 ± 0.51	1.85 ± 0.54	1.65 ± 0.48	0.135^a^

Continuous data displayed as mean ± SD if normally distributed or as median (IQR) if not normally distributed

The familywise error rate-adjusted Westfall-Young p values for differences in CD3 + T-cells (cells/μL), CD8 + T-cells (cells/μL), and CD19 + B-cells (cells/μL) at week 8 (11 hypothesis tests) were 0.136, 0.193, and 0.409, respectively

^a^Based on Student’s *t*-test

^b^Based on Wilcoxon (Mann–Whitney) rank sum test. * indicates significant differences in comparison to baseline at a *p* value < 0.05. Significant *p* values shown in bold

### Laboratory data: fatty acid profile

Table 5 displays selected fatty acids in plasma at baseline and after 8 weeks of the dietary intervention. Apart from docosahexaenoic acid, no statistically significant intergroup differences were found at week 8. Nevertheless, a clear trend was observed for some fatty acids, including arachidonic acid (319.05 ± 102.26 in the VD group vs. 373.77 ± 118.03 in individuals in the MD group). Plasma concentrations of alpha-linoleic acid and 11-eicosenoic acid significantly increased in those assigned to the VD group (Additional file 1: Figs. S4 and S5). Arachidonic acid significantly increased in those assigned to the MD group (Additional file 1: Fig. S6).

**Table 5:** Fatty acids in plasma at baseline and after 8 weeks of the dietary intervention

Laboratory value	Complete sample (*n* = 57)	Vegan group (*n* = 28)	Meat-rich group (*n* = 29)	*p* values
Baseline				
11-Eicosenoic acid (µmol/l)	10.55 ± 2.75	10.64 ± 3.19	10.47 ± 2.32	0.816^a^
Alpha-linolenic acid (µmol/l)	25.10 ± 11.18	24.15 ± 11.60	24.70 (16.68)	0.555^b^
Arachidic acid (µmol/l)	18.80 ± 3.93	18.86 ± 3.93	18.74 ± 3.99	0.911^a^
Arachidonic acid (µmol/l)	301.01 (130.38)	309.97 (146.22)	289.73 (120.12)	0.643^b^
Docosahexaenoic acid (µmol/l)	81.30 (37.33)	73.41 (50.83)	88.42 ± 23.34	0.355^b^
Gamma-linolenic acid (µmol/l)	9.91 (9.77)	10.14 (13.11)	11.44 ± 6.19	0.483^b^
Linoleic acid (µmol/l)	1290.04 (1114.61)	1388.86 (1110.64)	1218 (1097.80)	0.678^b^
Oleic acid (µmol/l)	857.32 (587.03)	974.66 ± 455.60	952.15 ± 337.28	0.833^a^
Week 8				
11-Eicosenoic acid (µmol/l)	12.33 (4.37)**	14.19 ± 4.45**	11.99 (3.59)*	0.055^b^
Alpha-linolenic acid (µmol/l)	25.31 (19.60)	28.51 (29.68)*	24.20 ± 10.91	0.100^b^
Arachidic acid (µmol/l)	18.44 ± 4.02	18.70 ± 4.18	18.19 ± 3.92	0.637^a^
Arachidonic acid (µmol/l)	346.89 ± 113.00*	319.05 ± 102.26	373.77 ± 118.03*	0.067^a^
Docosahexaenoic acid (µmol/l)	72.48 (32.05)*	66.19 ± 23.14*	82.92 (30.67)	**0.029**^b^
Gamma-linolenic acid (µmol/l)	11.88 (7.83)	13.21 ± 7.56	11.88 (6.36)	0.962^b^
Linoleic acid (µmol/l)	1147.03 (656.75)	1311.16 ± 582.61	1090.30 (570.18)	0.453^b^
Oleic acid (µmol/l)	811.72 (465.77)	941.67 ± 401.18	838.12 ± 264.63	0.253^a^

Continuous data displayed as mean ± SD if normally distributed or as median (IQR) if not normally distributed. Significant *p* values shown in bold. * indicates significant differences in comparison to baseline at a *p* value < 0.05; ** indicates significant differences in comparison to baseline at a *p* value < 0.001

The familywise error rate-adjusted Westfall-Young *p* value for the intergroup difference in docosahexaenoic acid at week 8 (16 hypothesis tests) was 0.071

^a^Based on Student’s *t*-test

^b^Based on Wilcoxon (Mann–Whitney) rank sum test

### Nutrient intake data

Table 6 displays nutrient intake data at baseline and during week 8 of the dietary intervention. Nutrient intake data from this study has been reported and discussed earlier in detail [17], but was re-calculated here based on the slightly smaller study sample in this sub-analysis. We observed no significant energy intake differences at baseline and at week 8 of the study. As such, reporting of energy-adjusted nutrient intakes was not deemed necessary. Significant intergroup differences were found for protein, fiber, calcium, sodium, phosphorus, and zinc intake at week 8. The Westfall-Young *p* values for these nutrients were as follows: 0.033, 0.015, 0.029, 0.008, 0.013, and 0.003, respectively. In line with the study-specific dietary requirements, no major intake shifts for fat (and fat expressed as a percentage of total energy intake) was observed.

**Table 6:** Nutrient intakes at baseline and after 8 weeks of the dietary intervention

Nutrient	Complete sample (*n* = 57)	Vegan group (*n* = 28)	Meat-rich group (*n* = 29)	*p* values
Baseline				
Energy (kcal/d)	2338.67 (794.67)	2388.06 ± 707.15	2426.12 ± 743.44	0.844^a^
Fat (g/d)	84.10 (40.56)	81.33 (47.07)	88.42 ± 32.58	0.836^b^
Carbohydrate (g/d)	265.50 (108.38)	264.83 ± 95.52	271.09 (96.02)	0.434^b^
Protein (g/d)	88.28 (49.94)	97.53 ± 29.93	90.79 ± 26.43	0.371^a^
Fiber (g/d)	28.41 ± 12.64	26.21 ± 12.60	30.53 ± 12.53	0.201^a^
Moisture (ml/d)	2716.68 ± 901.59	2962.77 ± 926.66	2479.08 ± 823.81	**0.042**^a^
Calcium (mg/d)	663.77 (329.00)	700.09 (368.78)	679.89 ± 231.60	0.911^b^
Potassium (mg/d)	2144.23 (1268.23)	2147.87 (1198.78)	2178.28 (738.81)	0.610^b^
Sodium (mg/d)	2388.61 (1077.30)	2450.69 (1378.42)	2369.61 ± 897.54	0.407^b^
Iron (mg/d)	9.16 (6.82)	8.79 (6.30)	10.21 ± 4.68	0.917^b^
Magnesium (mg/d)	273.19 (170.12)	234.18 (138.20)	284.45 (215.26)	0.987^b^
Phosphorus (mg/d)	944.62 (656.36)	884.32 (700.01)	1028.19 ± 363.75	0.823^b^
Zinc (mg/d)	8.19 (5.60)	8.77 ± 4.48	8.19 (5.40)	0.949^b^
Week 8				
Energy (kcal/d)	2382.67 (1086.67)	2293.67 (975.5)	2453.51 ± 873.35	0.898^b^
Fat (g/d)	82.14 (50.49)	83.38 ± 29.91	83.95 (39.24)	0.285^b^
Carbohydrate (g/d)	265.38 (119.50)	272.20 (85.44)*	267.41 ± 109.37	0.196^b^
Protein (g/d)	86.16 (47.19)	76.66 (36.95)*	107 (46.92)*	**0.005**^b^
Fiber (g/d)	30.80 (15.95)*	36.07 (17.20)**	23.12 (12.58)	** < 0.001**^b^
Moisture (ml/d)	3216.68 ± 1141.39*	3283.93 ± 1068.81*	2910.33 (1030.39)*	0.322^a^
Calcium (mg/d)	567.63 (424.96)	539.23 (213.38)	720 (549.42)*	**0.006**^b^
Potassium (mg/d)	2210.77 (829.92)	2369 (892.89)	2169.48 (829.92)	0.534^b^
Sodium (mg/d)	2369 (1914.65)	2012.40 (971.51)	3263.28 ± 1519.87*	**0.006**^b^
Iron (mg/d)	9.22 (5.33)	9.08 (5.89)	9.26 (4.48)	0.924^b^
Magnesium (mg/d)	298.59 (159.23)*	323.62 (133.87)*	256.88 (152.02)	0.058^b^
Phosphorus (mg/d)	862.94 (687.58)	727.84 (502.22)*	1192.18 ± 556.67	**0.005**^b^
Zinc (mg/d)	6.91 (5.15)	5.03 (2.87)*	10.63 ± 5.42*	** < 0.001**^b^

Continuous data displayed as mean ± SD if normally distributed or as median (IQR) if not normally distributed. The familywise error rate-adjusted Westfall-Young *p* values for differences in protein, fiber, calcium, sodium, phosphorus, and zinc intake at week 8 (13 hypothesis tests) were 0.033, 0.015, 0.029, 0.008, 0.013, and 0.003, respectively. * indicates significant differences in comparison to baseline at a *p* value < 0.05; ** indicates significant differences in comparison to baseline at a *p* value < 0.001

Significant *p* values shown in bold

^a^Based on Student’s *t*-test

^b^Based on Wilcoxon (Mann–Whitney) rank sum test

### Plasma amino acids

Table 7 displays the participants’ plasma amino acid status at baseline and during week 8 of the dietary intervention. No significant baseline differences were observed between the two groups. At week 8, however, significant between-group differences were found for alanine, betaine, and valine. Participants on a meat-rich diet had significantly higher valine levels, whereas they also had significantly lower alanine levels. When taking the familywise error rate-adjusted Westfall-Young *p* values for the intergroup differences in these amino acids into account, these differences were no longer significant (*p* = 0.132, 0.237, and 0.135, respectively). As for the pre-post differences, we observed significant decreases for the following plasma amino acids in the VD group: cysteine, leucine/isoleucine, lysine, tryptophan, valine.

**Table 7:** Plasma amino acids in µM at baseline and after 8 weeks of the dietary intervention

Nutrient	Complete sample (*n* = 57)	Vegan group (*n* = 28)	Meat-rich group (*n* = 29)	*p* values
Baseline				
Alanine (µM)	55.55 ± 13.97	56.62 ± 16.41	54.51 ± 11.33	0.574^a^
Arginine (µM)	54.80 (17.60)	57.16 ± 10.20	52.20 ± 15.89	0.169^a^
Asparagine (µM)	91.80 (17.80)	92.40 (17.05)	90.88 ± 14.75	0.443^b^
Aspartic acid (µM)	12.10 ± 2.58	11.95 ± 2.16	12.26 ± 2.95	0.651^a^
Betaine (µM)	24.10 (10.70)	23.85 (11.05)	24.25 ± 9.01	0.725^b^
Choline (µM)	27.91 ± 5.35	28.31 ± 5.29	27.52 ± 5.48	0.582^a^
Cysteine (µM)	513.00 (117.00)	515.00 (98.00)	515.07 ± 89.28	0.725^b^
Glutamic acid (µM)	18.90 (10.00)	19.10 (10.25)	19.83 ± 7.00	0.549^b^
Glutamine (µM)	353.30 ± 40.41	363.21 ± 43.01	343.72 ± 35.91	0.068^a^
Glycine (µM)	284.33 ± 65.70	300.54 ± 56.85	268.69 ± 70.71	0.067^a^
Histidine (µM)	47.40 (10.60)	48.25 ± 6.50	44.90 (9.00)	0.247^b^
Isoleucine 2 (µM)	62.32 ± 17.02	61.96 ± 16.12	62.10 (19.80)	0.981^b^
Leucine/isoleucine (µM)	146.32 ± 35.78	146.64 ± 33.62	146.02 ± 38.34	0.948^a^
Lysine (µM)	196.51 ± 37.00	202.46 ± 40.89	190.76 ± 32.49	0.236^a^
Methionine (µM)	16.90 (13.00)	16.15 (16.25)	17.20 (7.50)	0.805^b^
Phenylalanine (µM)	116.96 ± 20	117.98 ± 17.21	115.97 ± 22.63	0.709^a^
Proline (µM)	215.00 (106.00)	247.43 ± 84.56	203.00 (69.00)	0.185^b^
Serine (µM)	63.20 (21.50)	65.65 (21.40)	64.82 ± 15.07	0.792^b^
Taurine (µM)	160.19 ± 46.38	167.46 ± 47.29	153.17 ± 45.19	0.249^a^
Threonine (µM)	164.00 (50.00)	171.57 ± 36.80	159.00 (39.00)	0.241^b^
Tryptophan (µM)	114.13 ± 21.54	117.56 ± 21.14	110.81 ± 21.77	0.241^a^
Tyrosine (µM)	41.05 ± 10.71	40.25 ± 10.16	41.82 ± 11.34	0.586^a^
Valine (µM)	369.07 ± 70.59	366.07 ± 62.99	371.97 ± 78.25	0.756^a^
Week 8				
Alanine (µM)	42.40 (21.80)*	49.35 (27.40)	39.40 (12.10)**	**0.034**^b^
Arginine (µM)	55.59 ± 11.12	54.95 (7.75)	54.80 ± 12.94	0.508^b^
Asparagine (µM)	89.72 ± 20.36	93.63 ± 19.12	85.95 ± 21.13	0.157^a^
Aspartic acid (µM)	9.68 (5.52)**	10.83 ± 2.75*	10.39 ± 3.75*	0.615^a^
Betaine (µM)	26.99 ± 11.25	30.61 ± 11.92*	23.49 ± 9.51	**0.016**^a^
Choline (µM)	27.70 (15.50)	27.85 ± 8.71	27.10 (15.00)	0.278^b^
Cysteine (µM)	429 (220.00)**	395.43 ± 137.00**	393 (232.00)**	0.643^b^
Glutamic acid (µM)	17.40 (7.50)*	18.00 (7.20)	17.00 (8.60)	0.376^b^
Glutamine (µM)	352.49 ± 60.49	360.50 (47.50)	337.79 ± 61.81	0.074^b^
Glycine (µM)	244.32 ± 98.43*	261.72 ± 105.57	227.52 ± 89.64*	0.192^a^
Histidine (µM)	49.56 ± 11.01	49.61 ± 8.48	49.51 ± 13.16	0.949^a^
Isoleucine 2 (µM)	78.70 (25.40)**	77.05 ± 23.15**	75.00 (22.40)**	0.867^b^
Leucine/isoleucine (µM)	130 (48.00)	131.71 ± 30.39*	135.00 (60.00)	0.185^b^
Lysine (µM)	148.38 ± 37.76**	156.98 ± 35.50**	140.08 ± 38.61**	0.091^a^
Methionine (µM)	35.50 (18.20)**	35.13 ± 15.44*	35.50 (16.30)*	0.678^b^
Phenylalanine (µM)	114 (23.00)	113.44 ± 20.90	114.00 (30.00)	0.358^b^
Proline (µM)	193 (88.00)	195.50 (99.50)	204.64 ± 73.69	0.463^b^
Serine (µM)	51.10 ± 13.03**	52.54 ± 12.90**	53.90 (14.40)**	0.534^b^
Taurine (µM)	149 (51.00)	149.30 ± 40.14*	143.84 ± 43.01	0.549^a^
Threonine (µM)	161.00 (45.00)	153.00 (61.00)	163.00 (34.00)	0.598^b^
Tryptophan (µM)	82.80 (58.60)**	85.44 ± 30.27**	94.30 (60.10)*	0.750^b^
Tyrosine (µM)	46.90 (13.30)*	45.73 ± 12.13*	47.10 (10.40)*	0.241^b^
Valine (µM)	359.86 ± 84.01	329.96 ± 72.04*	388.72 ± 85.76	**0.007**^a^

Continuous data displayed as mean ± SD if normally distributed or as median (IQR) if not normally distributed. * indicates significant differences in comparison to baseline at a *p* value < 0.05; ** indicates significant differences in comparison to baseline at a *p* value < 0.001

The familywise error rate-adjusted Westfall-Young *p* value for the intergroup difference in alanine, betaine, and valine at week 8 (23 hypothesis tests) were 0.132, 0.237, and 0.135, respectively

Significant *p* values shown in bold

^a^Based on Student’s *t*-test

^b^Based on Wilcoxon (Mann–Whitney) rank sum test

### Immunoglobulin levels

Table 8 displays the participants’ immunoglobulin levels at baseline and during week 8 of the dietary intervention. Results suggested no significant between-group differences at baseline and the end of the trial.

**Table 8:** Immunoglobulin levels at baseline and after 8 weeks of the dietary intervention

Nutrient	Complete sample (*n* = 57)	Vegan group (*n* = 28)	Meat-rich group (*n* = 29)	*p* values
Baseline				
IgA (g/L)	1.66 ± 0.75	1.68 ± 0.71	1.63 ± 0.79	0.830^a^
IgE (kU/L)	28.30 (86)	39.55 (86.35)	28.20 (50.80)	0.439^b^
IgG (g/L)	11.10 ± 1.91	11.31 ± 1.75	10.89 ± 2.06	0.414^a^
IgM (g/L)	0.96 (0.55)	0.86 (0.81)	0.99 (0.53)	0.955^b^
Week 8				
IgA (g/L)	1.64 ± 0.72	1.68 ± 0.70	1.60 ± 0.75	0.690^a^
IgE (kU/L)	32.50 (82.50)*	40.35 (89.40)	28.70 (79.80)*	0.330^b^
IgG (g/L)	10.72 ± 1.74**	10.87 ± 1.68*	10.57 ± 1.81*	0.518^a^
IgM (g/L)	0.96 (0.57)	0.91 (0.88)	0.96 (0.43)	0.955^b^

Continuous data displayed as mean ± SD if normally distributed or as median (IQR) if not normally distributed. * indicates significant differences in comparison to baseline at a *p* value < 0.05; ** indicates significant differences in comparison to baseline at a *p* value < 0.001

^a^Based on Student’s *t*-test

^b^Based on Wilcoxon (Mann–Whitney) rank sum test

### Correlation analyses

Additional file 1: Table S2 shows associations between nutrient intake data and hemogram parameters at week 8 of the study. Significant inverse associations were found between fiber intake and the lymphocyte count (*r*_*s*_ = − 0.32, *p* = 0.016), the white blood cell count (*r*_*p*_ = − 0.36, *p* = 0.005), and the monocyte count (*r*_*p*_ = − 0.30, *p* = 0.024). Platelet counts were inversely correlated with both magnesium and iron intakes. Additional file 1: Table S3 shows associations between selected laboratory parameters and hemogram parameters at week 8 of the study. Copper levels were significantly associated with the platelet count (*r*_*s*_ = 0.31, *p* = 0.019) and the white blood cell count (*r*_*s*_ = 0.30,* p* = 0.026).

In a similar style, Additional file 1: Table S4 shows correlations between plasma fatty acids and hemogram parameters at week 8 of the study. Results suggested an inverse association between platelet counts and levels of eicosenoic acid serum (*r*_*s*_ = − 0.32,* p* = 0.014). Additional file 1: Table S5 displays correlations between plasma amino acids and hemogram parameters at week 8 of the study. Higher plasma taurine levels were associated with higher platelet counts and higher lymphocyte count, respectively (*r*_*p*_ = 0.29, *p* = 0.031 and *r*_*s*_ = 0.28, *p* = 0.032, respectively). While blood cell counts were also inversely correlated with cysteine and glycine levels. In light of our previous findings [16], we also correlated end-to-baseline differences of several amino acids (leucine/isoleucine, isoleucine, leucine, and valine) with end-to-baseline difference in the examined cell counts (Additional file 1: Table S6). Results suggested positive associations between said differences of leucine/isoleucine and end-to-baseline differences of lymphocytes (*r*_*s*_ = 0.27, *p* = 0.040), white blood cells (*r*_*s*_ = 0.34, *p* = 0.010), and monocytes (*r*_*s*_ = 0.35, *p* = 0.008). Results are visualized in Additional file 1: Fig. S7. No associations were found for leucine and valine.

### rm-ANCOVA results

Results of the conducted rm-ANOVA are shown in Table 9. No significant effects were observed for all cell lines. A closer inspection of the pooled within-subject covariance matrix in all four models casted doubt on the validity of the required compound symmetry assumption (as assessed with Huynh–Feldt’s epsilon and Greenhouse–Geisser’s epsilon). We thus decided to expand the analysis and ran mixed model repeated measures.

**Table 9:** Comparison of platelet, neutrophil, leukocyte, and lymphocyte counts across dietary groups. Results of the repeated measures ANOVA are based on values from week 0 (baseline), week 4, and week 8

	Baseline		Week 4		Week 8		*p* value (baseline–end)		
	Vegan diet	MR diet	Vegan diet	MR diet	Vegan diet	MR diet	Time	Diet	Time × diet
Lymphocytes	1.83 ± 0.45	1.83 (0.56)	1.80 ± 0.52	2.06 ± 0.55	1.80 ± 0.53	2.06 (0.74)	0.632	0.084	0.236
Neutrophils	2.48 (1.14)	2.75 (0.86)	2.23 (1.17)	2.82 ± 0.76	2.55 ± 0.63	2.93 ± 0.79	0.579	0.336	0.101
Platelets	233.29 ± 54.63	222.00 (89.00)	220.21 ± 53.35	243.21 ± 49.43	219.25 ± 49.79	222 (73.00)	0.133	0.226	0.059
White blood cells	5.35 ± 1.35	5.15 (1.39)	5.12 ± 1.08	5.7 ± 1.02	5.43 ± 1.10	5.39 (1.92)	0.984	0.051	0.070

All cell lines in thousand/µL. This table shows unadjusted *p* values. No significant effects were observed. A closer inspection of the pooled within-subject covariance matrix in all four models casted doubt on the validity of the required compound symmetry assumption (as assessed with Huynh–Feldt’s epsilon and Greenhouse–Geisser’s epsilon)

### MMRM results

**Fig. 5:**
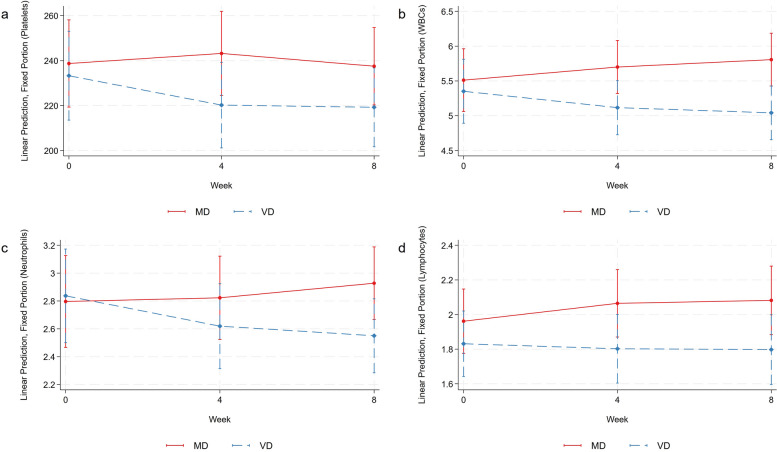
Mixed model repeated measures results. **a** depicts the platelet count (in thousand/µL); **b** depicts the leukocyte count (in thousand/µL); **c** depicts the neutrophil count (in thousand/µL); and **d** depicts the lymphocyte count (in thousand/µL)

Results of MMRMs were visualized using Stata’s marginsplot function and are depicted in Fig. 5. Stata’s contrast command with the diet-time interaction term was used for a joint test of the interaction including main effects. Results for the interaction term were as follows: *p* = 0.096 for the platelet count, *p* = 0.07 for the white blood cell count, *p* = 0.66 for the neutrophil count, and *p* = 0.295 for the lymphocyte count. While some trends are clearly visible in Fig. 5, no significant diet-time interaction terms were found. Follow-up tests were performed to determine whether any changes in the examined blood cell lines were simply due to the diet factor (e.g., VD vs. base). Here, a significant contrast of marginal linear predictions was observed for the leukocyte count (contrast: − 0.50 (95% CI: − 0.99–(− 0.01)), *p* = 0.046).

## Discussion

The present study investigated the effects of an isocaloric VD on whole blood count parameters, lymphocyte subsets, and other systemic inflammatory markers after an 8-week dietary intervention in comparison to a MD. Significant between-group differences were found, including a lower white blood cell and lymphocyte count in vegans. In addition, there was a trend towards lower neutrophil counts in vegans. The pre-post comparison revealed significantly lower platelet counts in vegans. Differences in mean/median change scores were significant for the neutrophil and platelet count. Immunophenotyping revealed significant between-group differences in the number of CD3^+^ T-cells, CD8^+^ T-cells, and CD19^+^ B-cells after 8 weeks. The number of CD19^+^ B-cells significantly decreased in the vegan group.

In the past decades, very few studies have specifically investigated blood cell counts in vegetarians and vegans [29]. Randomized dietary intervention studies in this field are scarce, as most of the evidence stems from cross-sectional investigations. The largest cross-sectional analysis was performed by Tong et al. and is based on data from the UK Biobank [29]. Analyzing data from more than 450,000 participants, Tong et al. observed lower white blood cell counts in white British vegetarians and vegans as compared to regular meat eaters (6.22 × 10^9^ cells/L (95% CI: 6.01, 6.43 × 10^9^ cells/L) vs. 7.02 × 10^9^ cells/L (95% CI: 7.01, 7.03 × 10^9^ cells/L)). This finding was largely in accordance with previous smaller studies by Haddad et al. [30], Obeid et al. [31], and Pongstaporn and Bunyaratavej [32], who investigated blood cell counts in different populations worldwide. As for the platelets, Tong et al. reported significantly lower counts in vegans as compared to omnivores (238.2 × 10^9^ cells/L; 95% CI: 232.5, 243.9 × 10^9^ cells/L vs. 254.5 × 10^9^ cells/L; 95% CI: 254.2, 254.7 × 10^9^ cells/L). In vegetarians, however, mean platelet counts did not differ when contrasted to omnivores (258.2 × 10^9^ cells/L; 95% CI: 256.8, 259.7 × 10^9^ cells/L). Neutrophils, lymphocytes, monocytes, eosinophils, and basophil counts appeared similar across the investigated diet groups [29].

Haddad et al. compared young Loma Linda-based vegans with non-vegetarians in a small comparative cross-sectional study including *n* = 45 participants [30]. Significantly lower numbers of platelets were found in vegans as compared to non-vegetarians (235 ± 60 × 10^9^/L vs. 270 ± 55 × 10^9^/L). Similarly, both groups differed with regard to the white blood cell count (4.96 ± 0.91 × 10^9^/L vs. 5.83 ± 1.51 × 10^9^/L) and the lymphocyte count (1.56 ± 0.39 × 10^9^/L vs. 1.90 ± 0.59 × 10^9^/L).

A German study compared blood cell counts between semi-vegetarians, lacto-ovo-vegetarians, and vegans [31]. Notably, median lymphocyte counts differed significantly between vegans (1.51 (1.08–2.37) and lacto-ovo-vegetarians (1.83 (1.26–2.76) but not between vegans and semi-vegetarians. No significant between-group differences were found for platelet counts. Finally, Nazarewicz compared selected blood count parameters between vegetarians and non-vegetarians [33]. Restricted to *n* = 41 participants, the author reported lower neutrophil counts in vegetarians as compared to non-vegetarians (3.33 G/l vs. 4.72 G/L) [33].

All aforementioned studies compared blood count parameters between different dietary patterns in diverse populations and in a comparative cross-sectional manner [29–33]. Subtle differences appeared in all studies, of which many align with our results. Blood cell count alterations subsequent to dietary interventions, however, have been rarely addressed in interventional studies.

One example for a non-controlled study is a 21-day Daniel Fast intervention by Bloomer et al., which centered around a 3-week quasi-vegan ad libitum food intake period that excluded animal products *and* preservatives while focusing on whole grains, fruits, vegetables, legumes, nuts, and seeds [34]. Subsequent to this dietary intervention, mean white blood cell count significantly decreased in a pre- vs. post-comparison (5.7 ± 0.2 vs. 5.0 ± 0.2 × 10^3^/μL) in *n* = 43 participants. Apart from the Bloomer study and the study by Link et al. [1, 34], only few randomized trials in this specific area exist. Our herein presented study thus fills a gap and allows for new insights into the anti-inflammatory effects of a VD. Our findings further suggest that diet-specific effects beyond caloric restriction could play a role.

A high white blood cell count has been repeatedly proposed as an early inflammation marker [29, 35, 36]. The total platelet count—which may be also affected by diet as discussed earlier—is also believed to be involved in chronic inflammation [29, 35]. As reviewed by Scherlinger et al., platelets interact with endothelial cells and white blood cells, produce soluble factors, and promote an inflammatory phenotype which may contribute to immune-mediated inflammatory diseases [37].

The fact that three blood cell lines (granulocytes, lymphocytes, and platelets) may be affected by diet is of utmost importance and could be of high value for precision nutrition strategies in many autoimmune and inflammatory disorders [3]. A recent systematic review and meta-analysis by Craddock et al. underscored these findings [3]. The exact mechanisms underlying the potential anti-inflammatory effects of plant-based diets and the mediators of the frequently observed lower blood cell counts in vegans, however, have been poorly understood. Lower intakes and/or serum levels of various nutrients have been discussed as potential causes for the lower counts of several blood cell types in vegetarians and vegans [29]. Examples include iron [29, 38] (mostly restricted to females, depending on the population [39, 40]), zinc [29] (a catalyst in iron metabolism [41]), vitamin A [29] (which is involved in normal cellular proliferation and differentiation processes [42, 43], and which may fall short on a VD [18, 19]), vitamin B12 [29] (with particular regard to erythropoiesis [44]), and branched-chain amino acids such as leucine, isoleucine, and valine [16]. Notably, higher levels of potentially anti-inflammatory nutrients, such as fiber and vitamin C, and its associated favorable gut microbiome alterations, could also play a pivotal role in this context [45, 46]. Conversely, vegans usually consume fewer pro-inflammatory saturated fatty acids [47], which have been shown to produce an inflammatory response through the activation of TLR4 signaling in the hypothalamus [48], and which increase pro-inflammatory pathways linked to an increased tryptophan to kynurenine conversion [49]. A more favorable ratio of n-6 to n-3 fatty acids and an increased intake of anti-inflammatory antioxidants have also been attributed to the anti-inflammatory effects of a VD [3, 10, 11]. Additional explanations include a reduced exposure to food additives and preservatives in plant-based diets, which may decrease the white blood cell count [34, 50, 51]. Finally, vegans were shown to have lower taurine levels which may also be important in the role of lymphoproliferation [52, 53].

Investigating all those potential explanations and mechanisms in a single study remains difficult. In our study, we focused on nutrient intake data and a series of laboratory markers as potential factors associated with the obtained results. Our analysis points at a potential role for fiber, which was inversely associated with lymphocyte counts (*r*_*s*_ = − 0.32, *p* = 0.016), white blood cell counts (*r*_*p*_ = − 0.36, *p* = 0.005), and monocyte counts (*r*_*p*_ = − 0.30, *p* = 0.024) after 8 weeks. In accordance with the predecessor study [16], we again found positive associations between end-to-baseline differences of leucine/isoleucine and end-to-baseline differences of lymphocytes (*r*_*s*_ = 0.27, *p* = 0.040), white blood cells (*r*_*s*_ = 0.34, *p* = 0.010), and monocytes (*r*_*s*_ = 0.35, *p* = 0.008). Taken together, these findings suggest that adoption of a VD elicits health-promoting changes in immune cell composition in just 4 weeks and that these modifications are sustained for at least to 8 weeks.

Our study is unique in a way that both dietary interventions were isoenergetic. Participants were instructed to avoid weight loss to *clinically* adjust for this important confounder. The herein presented results suggest that explanations other than body weight are involved in the potential immunomodulatory effects of vegan diets. Due to the body weight adjustment, our results are difficult to contrast to cross-sectional or non-interventional studies. Dietary intakes in vegans in our study might differ from free-living and community-dwelling vegans. The vegan arm in our study was designed with an isocaloric character in mind, which implied that some participants purposely consumed higher amounts of nuts, oils, or granola bars to achieve the daily energy intake target of 1800–2000 kcal/d. Thus, the macronutrient distribution observed here might not necessarily align with previous studies in vegans [54].

The present study allows for new insights into the anti-inflammatory potential of plant-based nutrition and reiterates our previous findings [16]. In addition, the study doubled the duration of the predecessor study and emphasizes that a dietary intervention may also affect lymphocyte levels. Lymphocytes have a much longer lifespan than granulocytes and we hypothesized that changes in lymphocyte counts could be more adequately captured with a study duration of 8 weeks [55, 56]. The obtained results are of high translational value and lay the foundation for additional follow-up studies in patients suffering from autoimmune disorders or chronic low-grade inflammation.

### Strengths and weaknesses of the present study

The present study has strengths and weaknesses that warrant further discussion. As for the strengths, the study reports a controlled dietary intervention with an adequate duration (8 weeks) to sufficiently capture hemogram alterations. Dietary intakes were assessed with a 3-day weighed food diary following established procedures [17, 18]. Benefiting from our previous experience [16], we improved and intensified the employed dietary counseling strategy and included both individual and group sessions in this follow-up study. An added strength of the study is the comprehensive confounder and mediator assessment by the inclusion of numerous covariates (e.g., physical activity, social status, supplementation). As for the weaknesses, it is important to emphasize that the study was performed in the pollen season between the months of April and June. The immune system is known to be affected by seasonal changes [55, 57]; however, there is no reason to suspect that this should vary by dietary group. Several participants had to be excluded from the analysis, though, mainly due to infections (e.g., one participant in the MD group suffered from pneumonia and one participant in the VD group from infectious mononucleosis). The VD prescribed here was not an ad libitum diet; to the contrary, some participants had to purposely consume higher amounts of nuts, oils, or granola bars to achieve their daily energy intake goal of 1800–2000 kcal/d. This may reduce the external validity of this study and may not precisely represent real-world dietary practices of free-living vegans; yet it allowed us to eliminate weight loss as a confounder. While unprocessed plant foods were encouraged in the VD group as reported earlier [17], participants were naïve to the VD, which requires considerable time and knowledge to adopt and master [17, 58]. As such, participants also selected meat analogs and wheat gluten-based meat replacement products [17], which should be considered when discussing the results of the precent study. Analytical approaches with single-factor/single-nutrient analyses (e.g., bivariate correlations using fiber, iron, and magnesium intake) have intrinsic limitations, as all aforementioned nutrients are a proxy of plant food intake. In the same direction, linear mixed ANCOVA models for selected exploratory (non-primary/non-secondary) outcomes (e.g., amino acid concentrations) instead of Wilcoxon rank sum tests or *t*-tests could have revealed interactions not captured with our statistical approach. Then again, we ran MMRMs for the primary and secondary outcome exactly because a closer inspection of the pooled within-subject covariance matrix in all four rm-ANCOVA models casted doubt on the validity of the required compound symmetry assumption. Finally, neural networks and other machine learning approaches could uncover multi-nutrient interactions that better describe the physiological compartment under study (plasma, cells).

Despite these limitations, our results substantially add to the existing literature on the effects of vegan dietary interventions on hemogram parameters and lymphoid composition. While of importance, environmental factors that could have affected the study outcomes were not fully considered in this trial (e.g., air temperature variability and other seasonal phenomena [57, 59]) and should be ideally considered in future studies.

## Conclusions

The present study suggests that a VD may be a complementary means to alter lymphoid composition. A short-term vegan dietary modification has subtle impacts on whole blood count parameters and circulating immune cells, even in healthy individuals. Our findings may have both implications for precision nutritional interventions as well as for public health nutrition strategies.

## Supplementary Information

Additional file 1. Tables S1–S6 and Figures S1–S7; Table S1 Changes in body weight over the course of the study: an overview; Table S2 Correlation analyses between nutrient intake data and selected hemogram parameters at week 8 of the study; Table S3 Correlation analyses between laboratory data and selected hemogram parameters at week 8 of the study; Table S4 Correlation analyses between serum fatty acids and selected hemogram parameters at week 8 of the study; Table S5 Correlation analyses between plasma amino acids and selected hemogram parameters at week 8 of the study; Table S6 Correlation analyses between changes in plasma amino acid levels (week 8 vs. baseline) and selected hemogram parameters (week 8 vs. baseline); Fig. S1 White blood cell count in thousand/µL by dietary group at baseline and after 4 and 8 weeks of the dietary intervention, respectively; Fig. S2 Neutrophil count by dietary group at baseline and after 4 and 8 weeks of the dietary intervention, respectively; Fig. S3 High-sensitive C-reactive protein (hs-CRP) levels in mg/l by dietary group at baseline and after 8 weeks of the dietary intervention; Fig. S4 Alpha-linoleic acid levels in µmol/l by dietary group at baseline and after 8 weeks of the dietary intervention; Fig. S5 11-Eicosenoic acid levels in µmol/l by dietary group at baseline and after 8 weeks of the dietary intervention; Fig. S6 Arachidonic acid levels in µmol/l by dietary group at baseline and after 8 weeks of the dietary intervention; Fig. S7 Correlations between end-to-baseline differences of various amino acids (leucine/isoleucine, isoleucine 2, lysine, and valine) and end-to-baseline difference in lymphocytes (panel A), white blood cells (panel B), and monocytes (panel C).

## Data Availability

The datasets used and analyzed for the current study are available from the corresponding author (Maximilian Andreas Storz) on reasonable request.

## Abbreviations

CRP: C-reactive protein
DRKS: Deutsches Register Klinischer Studien
HEPA: Health-enhancing physical activity
IPAQ-SF: International Physical Activity Questionnaire—Short Form
MASSS: MacArthur Scale of Subjective Social Status
MD: Meat-rich diet
MMRM: Mixed model repeated measures
RA: Rheumatoid arthritis
VD: Vegan diet
