# Supplementary material for: Impact of an eight-week isocaloric vegan dietary intervention on hemogram parameters and lymphocyte subsets: a randomized-controlled trial
Source: BMC Med. 2026 Jan 10;24:53. doi: 10.1186/s12916-025-04612-y (PMC12849501; doi:10.1186/s12916-025-04612-y)
Supplement: Supplementary file 1 — Additional file 1. Tables S1–S6 and Figures S1–S7; Table S1 Changes in body weight over the course of the study: an overview; Table S2 Correlation analyses between nutrient intake data and selected hemogram parameters at week 8 of the study; Table S3 Correlation analyses between laboratory data and selected hemogram parameters at week 8 of the study; Table S4 Correlation analyses between serum fatty acids and selected hemogram parameters at week 8 of the study; Table S5 Correlation analyses between plasma amino acids and selected hemogram parameters at week 8 of the study; Table S6 Correlation analyses between changes in plasma amino acid levels (week 8 vs. baseline) and selected hemogram parameters (week 8 vs. baseline); Fig. S1 White blood cell count in thousand/µL by dietary group at baseline and after 4 and 8 weeks of the dietary intervention, respectively; Fig. S2 Neutrophil count by dietary group at baseline and after 4 and 8 weeks of the dietary intervention, respectively; Fig. S3 High-sensitive C-reactive protein (hs-CRP) levels in mg/l by dietary group at baseline and after 8 weeks of the dietary intervention; Fig. S4 Alpha-linoleic acid levels in µmol/l by dietary group at baseline and after 8 weeks of the dietary intervention; Fig. S5 11-Eicosenoic acid levels in µmol/l by dietary group at baseline and after 8 weeks of the dietary intervention; Fig. S6 Arachidonic acid levels in µmol/l by dietary group at baseline and after 8 weeks of the dietary intervention; Fig. S7 Correlations between end-to-baseline differences of various amino acids (leucine/isoleucine, isoleucine 2, lysine, and valine) and end-to-baseline difference in lymphocytes (panel A), white blood cells (panel B), and monocytes (panel C). [file 12916_2025_4612_MOESM1_ESM.docx]

# Additional File 1

Additional File 1 with supplementary tables and figures for “Impact of an eight-week isocaloric vegan dietary intervention on hemogram parameters and lymphocyte subsets: a randomized-controlled trial” by Herter et al.

# Supplementary Tables

## Supplementary Table S1

Supplementary Table S1 title: Changes in body weight over the course of the study: an overview

| Variable | Complete sample (n=57) | Vegan group  (n=28) | Meat-rich group (n=29) | p-value |
| --- | --- | --- | --- | --- |
| Baseline | | | | |
| Weight (kg) | 74.45 ± 10 ^a^ | 74.58 ± 9.73 ^b^ | 74.33 ± 10.41 | 0.927 ^c^ |
| Week 4 | | | | |
| Weight (kg) | 74.05 ± 9.90 | 74.33 ± 9.54 | 73.79 ± 10.40 | 0.841 ^c^ |
| Week 8 | | | | |
| Weight (kg) | 73.28 ± 10.12 | 73.39 ± 9.99 | 73.18 ± 10.41 | 0.937 ^c^ |
| Δweight baseline vs. week eight (kg) | 1.32 ± 1.32 ^a^ | 1.50 ± 1.60 ^a^ | 1.15 ± 0.99 | 0.328 ^c^ |

Supplementary Table S1 legend: Continuous data displayed as mean ± SD if normally distributed or as median (IQR) if not normally distributed. ^a^ = based on 56 observations. ^b^ = based on n = 27 observations (no anthropometric data available for one person at baseline. ^c^ = based on Student's t-test analyses)

## Supplementary Table S2

Supplementary Table S2 title: Correlation analyses between nutrient intake data and selected hemogram parameters at week 8 of the study

| **Nutrients** | Platelets  (thousand/µL) | *p*-value | Neutrophils  (thousand/µL) | *p*-value | Lymphocytes (thousand/µL) | *p*-value | White blood cells (thousand/µL) | *p*-value | Monocytes (thousand/µL) | *p*-value |
| --- | --- | --- | --- | --- | --- | --- | --- | --- | --- | --- |
| Calcium (mg/d) | r_s_ = -0.08 | 0.568 | r_s_ = 0.10 | 0.472 | r_s_ = -0.05 | 0.703 | r_s_ = 0.01 | 0.956 | r_s_ = -0.04 | 0.781 |
| Carbohydrate (g/d) | r_s_ = -0.30 | **0.023** | r_s_ = -0.12 | 0.362 | r_p_ = -0.20 | 0.128 | r_s_ = -0.21 | 0.112 | r_p_ = -0.33 | **0.013** |
| Energy (kcal/d) | r_s_ = -0.27 | **0.041** | r_s_ = -0.10 | 0.455 | r_s_ = -0.21 | 0.115 | r_s_ = -0.21 | 0.123 | r_s_ = -0.17 | 0.195 |
| Fat (g/d) | r_s_ = -0.19 | 0.157 | r_s_ = 0.02 | 0.866 | r_s_ = -0.18 | 0.176 | r_s_ = -0.11 | 0.400 | r_s_ = -0.03 | 0.852 |
| Fiber (g/d) | r_s_ = -0.15 | 0.261 | r_s_ = -0.22 | 0.097 | r_s_ = -0.32 | **0.016** | r_p_ = -0.36 | **0.005** | r_p_ = -0.30 | **0.024** |
| Iron (mg/d) | r_s_ = -0.34 | **0.009** | r_s_ = -0.12 | 0.384 | r_s_ = -0.10 | 0.457 | r_s_ = -0.12 | 0.376 | r_s_ = 0.01 | 0.957 |
| Magnesium (mg/d) | r_s_ = -0.31 | **0.019** | r_s_ = -0.25 | 0.059 | r_s_ = -0.17 | 0.199 | r_s_ = -0.25 | 0.060 | r_s_ = 0.01 | 0.932 |
| Phosphorus (mg/d) | r_s_ = -0.19 | 0.156 | r_s_ = -0.04 | 0.763 | r_s_ = 0.05 | 0.700 | r_s_ = 0.00 | 0.991 | r_s_ = 0.13 | 0.344 |
| Potassium (mg/d) | r_s_ = -0.23 | 0.081 | r_s_ = -0.19 | 0.160 | r_s_ = -0.17 | 0.208 | r_s_ = -0.27 | 0.050 | r_s_ = 0.02 | 0.898 |
| Protein (g/d) | r_s_ = -0.19 | 0.166 | r_s_ = -0.04 | 0.785 | r_s_ = 0.01 | 0.927 | r_s_ = -0.03 | 0.825 | r_s_ = -0.03 | 0.836 |
| Sodium (mg/d) | r_s_ = -0.12 | 0.392 | r_s_ = 0.16 | 0.230 | r_s_ = 0.05 | 0.701 | r_s_ = 0.12 | 0.389 | r_s_ = -0.06 | 0.652 |

Supplementary Table S2: r_s_ = Spearman's Rho, based on Spearman's Rank Order Correlation; *r*_p_ = Pearson’s r, based on a Pearson product-moment correlation

## Supplementary Table S3

Supplementary Table S3 title: Correlation analyses between laboratory data and selected hemogram parameters at week 8 of the study

| **Laboratory values** | Platelets  (thousand/µL) | *p*-value | Neutrophils  (thousand/µL) | *p*-value | Lymphocytes (thousand/µL) | *p*-value | White blood cells (thousand/µL) | *p*-value | Monocytes (thousand/µL) | *p*-value |
| --- | --- | --- | --- | --- | --- | --- | --- | --- | --- | --- |
| Cholesterol (mg/dl) | r_s_ = 0.13 | 0.330 | r_s_ = 0.11 | 0.413 | r_s_ = 0.11 | 0.421 | r_s_ = 0.13 | 0.324 | r_s_ = -0.20 | 0.139 |
| Copper (µg /dl) | r_s_ = 0.31 | **0.019** | r_s_ = 0.15 | 0.267 | r_s_ = 0.24 | 0.076 | r_s_ = 0.30 | **0.026** | r_s_ = 0.25 | 0.058 |
| Ferritin (ng/ml) | r_s_ = -0.11 | 0.411 | r_s_ = -0.05 | 0.706 | r_s_ = 0.15 | 0.274 | r_s_ = 0.09 | 0.514 | r_s_ = -0.07 | 0.597 |
| Folic acid (ng/ml) | r_s_ = -0.11 | 0.431 | r_s_ = -0.03 | 0.837 | r_s_ = 0.05 | 0.706 | r_s_ = -0.01 | 0.967 | r_s_ = -0.03 | 0.850 |
| HbA1C (%) | r_s_ = -0.04 | 0.789 | r_s_ = -0.23 | 0.079 | r_s_ = -0.25 | 0.061 | r_s_ = -0.31 | **0.020** | r_s_ = -0.16 | 0.232 |
| High-density lipoprotein (mg/dl) | r_s_ = 0.194 | 0.149 | r_s_ = 0.17 | 0.216 | r_s_ = 0.10 | 0.439 | r_s_ = 0.23 | 0.092 | r_s_ = 0.01 | 0.941 |
| High-sensitive CRP (mg/l) | r_s_ = 0.18 | 0.171 | r_s_ = 0.10 | 0.473 | r_s_ = 0.09 | 0.526 | r_s_ = 0.13 | 0.334 | r_s_ = 0.07 | 0.602 |
| Holotranscobalamin (pmol/l) | r_s_ = -0.11 | 0.411 | r_s_ = 0.09 | 0.525 | r_s_ = 0.18 | 0.190 | r_s_ = 0.09 | 0.501 | r_s_ = -0.15 | 0.282 |
| Iron (μg/dl) | r_s_ = 0.04 | 0.746 | r_s_ = -0.12 | 0.391 | r_s_ = 0.04 | 0.750 | r_s_ = -0.03 | 0.818 | r_s_ = -0.07 | 0.583 |
| Low-density lipoprotein (mg/dl) | r_s_ = -0.00 | 0.98 | r_s_ = 0.01 | 0.961 | r_s_ = 0.02 | 0.908 | r_s_ = -0.02 | 0.895 | r_s_ = -0.24 | 0.074 |
| Serum triglycerides (mg/l) | r_s_ = -0.15 | 0.265 | r_s_ = 0.21 | 0.123 | r_s_ = 0.02 | 0.860 | r_s_ = 0.16 | 0.238 | r_s_ = -0.08 | 0.563 |
| Transferrin (mg/dl) | r_s_ = 0.36 | **0.005** | r_s_ = 0.21 | 0.113 | r_s_ = -0.13 | 0.353 | r_s_ = 0.09 | 0.487 | r_s_ = 0.05 | 0.718 |
| Uric acid(mg/dl) | r_s_ = 0.03 | 0.836 | r_s_ = -0.16 | 0.230 | r_s_ = -0.03 | 0.822 | r_s_ = -0.14 | 0.314 | r_s_ = 0.09 | 0.500 |
| Vitamin B12 (pg/ml) | r_s_ = -0.04 | 0.786 | r_s_ = -0.01 | 0.939 | r_s_ = 0.09 | 0.503 | r_s_ = 0.03 | 0.821 | r_s_ = -0.00 | 0.991 |
| Zinc (µg /dl) | r_p_= 0.28 | **0.032** | r_s_ = -0.07 | 0.593 | r_p_ = 0.10 | 0.46 | r_s_ = 0.04 | 0.747 | r_s_ = -0.00 | 0.998 |

## Supplementary Table S4

Supplementary Table S4 title: Correlation analyses between serum fatty acids and selected hemogram parameters at week 8 of the study

| **Laboratory values** | Platelets  (thousand/µL) | *p*-value | Neutrophils  (thousand/µL) | *p*-value | Lymphocytes (thousand/µL) | *p*-value | White blood cells (thousand/µL) | *p*-value | Monocytes (thousand/µL) | *p*-value |
| --- | --- | --- | --- | --- | --- | --- | --- | --- | --- | --- |
| Alpha-Linolenic acid (µmol/l) | *r_s_* = -0.07 | 0.601 | *r_s_* = 0.13 | 0.326 | *r_s_* = 0.00 | 0.988 | *r_s_* = 0.09 | 0.488 | *r_s_* = -0.04 | 0.764 |
| Arachidic acid (µmol/l) | *r_s_* = -0.03 | 0.829 | *r_s_* = 0.06 | 0.647 | *r_s_* = -0.02 | 0.897 | *r_p_* = 0.04 | 0.753 | *r_s_* = -0.14 | 0.304 |
| Arachidonic acid (µmol/l) | *r_p_* = 0.21 | 0.114 | *r_s_* = -0.04 | 0.747 | *r_s_* = 0.12 | 0.376 | *r_p_* = 0.07 | 0.592 | *r_s_* = -0.19 | 0.147 |
| Docosahexaenoic acid (µmol/l) | *r_s_* = 0.26 | **0.047** | *r_s_* = 0.11 | 0.407 | *r_s_* = 0.23 | 0.087 | *r_s_* = 0.21 | 0.116 | *r_s_* = 0.03 | 0.851 |
| Eicosenoic acid (µmol/l) | *r_s_* = -0.32 | **0.014** | *r_s_* = 0.02 | 0.862 | *r_s_* = -0.17 | 0.196 | *r_s_* = -0.08 | 0.579 | *r_s_* = -0.03 | 0.815 |
| Gamma-Linolenic acid (µmol/l) | *r_s_* = -0.04 | 0.742 | *r_s_* = -0.11 | 0.400 | *r_s_* = -0.10 | 0.471 | *r_s_* = -0.17 | 0.209 | *r_s_* = -0.26 | 0.050 |
| Linoleic acid (µmol/l) | *rs* = -0.11 | 0.413 | *r_s_* = 0.05 | 0.687 | *r_s_* = -0.06 | 0.661 | *r_s_* = -0.03 | 0.817 | *r_s_* = -0.07 | 0.608 |
| Oleic acid (µmol/l) | *r_s_* = -0.17 | 0.206 | *r_s_* = -0.01 | 0.930 | *r_s_* = -0.08 | 0.550 | *r_s_* = -0.08 | 0.576 | *r_s_* = -0.03 | 0.836 |

Supplementary Table S4: r_s_ = Spearman's Rho, based on Spearman's Rank Order Correlation; *r*_p_ = Pearson’s r, based on a Pearson product-moment correlation

## Supplementary Table S5

Supplementary Table S5 title: Correlation analyses between plasma amino acids and selected hemogram parameters at week 8 of the study

| **Aminoacids** | Platelets  (thousand/µL) | *p*-value | Neutrophils  (thousand/µL) | *p*-value | Lymphocytes (thousand/µL) | *p*-value | White blood cells (thousand/µL) | *p*-value | Monocytes (thousand/µL) | *p*-value |
| --- | --- | --- | --- | --- | --- | --- | --- | --- | --- | --- |
| Alanine [µM] | *r_s_* =0.10 | 0.476 | *r_s_* = -0.14 | 0.316 | *r_s_* = -0.11 | 0.414 | *r_s_* = -0.18 | 0.180 | *r_s_* = -0.02 | 0.860 |
| Arginine [µM] | *r_p_* = 0.32 | **0.014** | *r_s_* = -0.13 | 0.340 | *r_s_* = -0.05 | 0.688 | *r_p_* = -0.12 | 0.338 | *r_s_* = 0.03 | 0.821 |
| Asparagine [µM] | *r_p_* = 0.13 | 0.338 | *r_s_* = -0.27 | **0.043** | *r_s_* = -0.08 | 0.561 | *r_s_* = -0.26 | 0.050 | *r_s_* = -0.19 | 0.163 |
| Aspartic acid [µM] | *r_s_* = 0.38 | **0.004** | *r_s_* = 0.05 | 0.695 | *r_s_* = 0.11 | 0.405 | *r_s_* = 0.13 | 0.318 | *r_s_* = -0.03 | 0.835 |
| Betaine [µM] | *r_s_* = -0.19 | 0.150 | *r_s_* = -0.15 | 0.251 | *r_s_* = 0.09 | 0.527 | *r_s_* = -0.03 | 0.804 | *r_s_* = -0.07 | 0.613 |
| Choline [µM] | *r_s_* = -0.02 | 0.886 | *r_s_* = -0.27 | **0.044** | *r_s_* = -0.08 | 0.539 | *r_s_* = -0.23 | 0.085 | *r_s_* = -0.13 | 0.323 |
| Cysteine [µM] | *r_s_* = 0.04 | 0.762 | *r_s_* = -0.24 | 0.076 | *r_s_* = -0.23 | 0.088 | *r_s_* = -0.31 | **0.019** | *r_s_* = -0.25 | 0.059 |
| GlutamicAcid [µM] | *r_s_* = 0.05 | 0.689 | *r_s_* = 0.03 | 0.802 | *r_s_* = 0.13 | 0.348 | *r_s_* = 0.10 | 0.447 | *r_s_* = 0.09 | 0.491 |
| Glutamine [µM] | *r_p_* = 0.01 | 0.945 | *r_s_* = -0.23 | 0.088 | *r_s_* = 0.02 | 0.879 | *r_p_* = -0.13 | 0.350 | *r_p_* = -0.19 | 0.162 |
| Glycine [µM] | *r_s_ =* 0.06 | 0.634 | *r_s_* = -0.29 | **0.029** | *r_s_* = -0.13 | 0.332 | *r_p_* = -0.27 | **0.041** | *r_s_* = -0.22 | 0.101 |
| Histidine [µM] | *r_p_* = 0.15 | 0.274 | *r_s_* = -0.18 | 0.171 | *r_s_* = -0.02 | 0.899 | *r_s_* = -0.11 | 0.403 | *r_p_* = -0.25 | 0.059 |
| Isoleucine2 [µM] | *r_s_* = 0.00 | 0.977 | *r_s_* = -0.08 | 0.575 | *r_s_* = 0.23 | 0.085 | *r_s_* = 0.03 | 0.824 | *r_s_* = 0.07 | 0.588 |
| Leucine/Isoleucine [µM] | *r_s_* = 0.11 | 0.420 | *r_s_* = -0.15 | 0.268 | *r_s_* = 0.03 | 0.809 | *r_s_* = -0.12 | 0.371 | *r_s_* = -0.07 | 0.615 |
| Lysine [µM] | *rp* = -0.13 | 0.346 | *r_s_* = -0.12 | 0.358 | *r_s_* = -0.19 | 0.158 | *r_p_* = -0.18 | 0.170 | *r_s_* = -0.16 | 0.237 |
| Methionine [µM] | *r_s_* = 0.26 | **0.049** | *r_s_* = 0.05 | 0.686 | *r_s_* = 0.15 | **0.263** | *r_s_* = 0.18 | 0.177 | *r_s_* = 0.13 | 0.342 |
| Phenylalanine [µM] | *r_s_* = 0.21 | 0.113 | *r_s_* = -0.06 | 0.647 | *r_s_* = 0.18 | 0.185 | *r_s_* = 0.04 | 0.760 | *r_s_* = 0.00 | 0.999 |
| Proline [µM] | *r_s_* = -0.01 | 0.938 | *r_s_* = -0.14 | 0.288 | *r_s_* = -0.05 | 0.727 | *r_s_* = -0.11 | 0.426 | *r_s_* = -0.14 | 0.286 |
| Serine [µM] | *r_s_* = 0.13 | 0.328 | *r_s_* = -0.14 | 0.315 | *r_s_* = 0.01 | 0.958 | *r_s_* = -0.08 | 0.545 | *r_s_* = -0.02 | 0.880 |
| Taurine [µM] | *r_p_* = 0.29 | **0.031** | *r_s_* = 0.04 | 0.783 | *r_s_* = 0.28 | **0.032** | *r_s_* = 0.19 | 0.149 | *r_s_* = 0.03 | 0.842 |
| Threonine [µM] | *r_s_* = 0.30 | **0.022** | *r_s_* = -0.13 | 0.339 | *r_s_* = 0.13 | 0.352 | *r_s_* = 0.02 | 0.899 | *r_s_* = 0.058 | 0.669 |
| Tryptophan [µM] | *r_s_* = 0.11 | 0.409 | *r_s_* = -0.06 | 0.645 | *r_s_* = -0.06 | 0.646 | *r_s_* = -0.07 | 0.618 | *r_s_* = -0.13 | 0.319 |
| Tyrosine [µM] | *r_s_* = 0.21 | 0.109 | *r_s_* = -0.03 | 0.804 | *r_s_* = 0.12 | 0.381 | *r_s_* = -0.00 | 0.996 | *r_s_* = -0.055 | 0.687 |
| Valine [µM] | *r_p_* = 0.18 | 0.162 | *r_s_* = -0.03 | 0.803 | *r_s_* = 0.23 | 0.089 | *r_p_* = 0.09 | 0.506 | *r_s_* = 0.04 | 0.761 |

## Supplementary Table S6

Supplementary Table S6 title: Correlation analyses between changes in plasma amino acid levels (week 8 vs. baseline) and selected hemogram parameters (week 8 vs. baseline)

| **Aminoacids** | Δ Platelets  (thousand/µL) | *p*-value | Δ Neutrophils  (thousand/µL) | *p*-value | Δ Lymphocytes (thousand/µL) | *p*-value | Δ White blood cells (thousand/µL) | *p*-value | Δ Monocytes (thousand/µL) | *p*-value |
| --- | --- | --- | --- | --- | --- | --- | --- | --- | --- | --- |
| Δ Alanine | *r_s_* = -0.24 | 0.073 | *r_s_* = -0.27 | **0.043** | *r_p_* = -0.01 | 0.958 | *r_s_* = -0.25 | 0.061 | *r_s_* = -0.01 | 0.970 |
| Δ Arginine | *r_s_* = -0.03 | 0.814 | *r_s_* = -0.07 | 0.582 | *r_s_* = -0.04 | 0.741 | *r_s_* = -0.07 | 0.612 | *r_s_* = 0.13 | 0.326 |
| Δ Asparagine | *r_p_* = 0.01 | 0.913 | *r_s_* = 0.03 | 0.853 | *r_s_* = -0.04 | 0.779 | *r_s_* = 0.04 | 0.783 | *r_s_* = 0.17 | 0.199 |
| Δ Aspartic Acid | *r_s_* = 0.02 | 0.908 | *r_s_* = 0.05 | 0.730 | *r_s_* = 0.12 | 0.384 | *r_s_* = 0.05 | 0.686 | *r_s_* = 0.17 | 0.215 |
| Δ Betaine | *r_s_* = -0.00 | 0.994 | *r_s_* = -0.08 | 0.545 | *r_s_* = 0.07 | 0.588 | *r_s_* = -0.09 | 0.511 | *r_p_* = 0.11 | 0.420 |
| Δ Choline | *r_s_* = 0.12 | 0.388 | *r_s_* = 0.06 | 0.637 | *r_s_* = 0.05 | 0.709 | *r_s_* = 0.05 | 0.703 | *r_s_* = 0.20 | 0.134 |
| Δ Cysteine | *r_s_* = 0.17 | 0.200 | *r_s_* = 0.06 | 0.684 | *r_s_* = 0.01 | 0.933 | *r_s_* = 0.03 | 0.851 | *r_s_* = 0.14 | 0.293 |
| Δ GlutamicAcid | *r_p_* = -0.02 | 0.893 | *r_p_* = -0.19 | 0.152 | *r_p_*= 0.04 | 0.772 | *r_p_* = -0.13 | 0.349 | *r_p_* = -0.14 | 0.315 |
| Δ Glutamine | *r_p_*= -0.14 | 0.316 | *r_p_* = 0.16 | 0.221 | *r_p_* = 0.10 | 0.441 | *r_p_* = 0.16 | 0.242 | r_p_ = 0.15 | 0.268 |
| Δ Glycine | r_p_ = -0.16 | 0.244 | *r_s_* = 0.08 | 0.539 | *r_p_* = 0.00 | 0.983 | *r_p_* = 0.10 | 0.468 | *r_p_* = 0.07 | 0.583 |
| Δ Histidine | *rs* = 0.03 | 0.801 | *r_s_* = 0.11 | 0.430 | *r_p_* = 0.11 | 0.404 | *r_s_* = 0.11 | 0.430 | *r_p_* = 0.25 | 0.063 |
| Δ Isoleucine2 | *r_s_* = -0.17 | 0.198 | *r_s_* = -0.07 | 0.620 | *r_s_* = 0.22 | 0.106 | *r_s_* = 0.15 | 0.262 | *r_p_* = 0.07 | 0.612 |
| Δ Leucine/ isoleucine | *r_s_* = 0.11 | 0.428 | *r_s_* = 0.18 | 0.174 | *r_s_* = 0.27 | **0.040** | *r_s_* = 0.34 | **0.010** | *r_s_* = 0.35 | **0.008** |
| Δ Lysine | *r_p_* = -0.04 | 0.741 | *r_p_* = 0.24 | 0.072 | r_p_ = -0.08 | 0.561 | *r_p_* = 0.14 | 0.309 | *r_p_* = 0.02 | 0.856 |
| Δ Methionine | *r_s_* = 0.18 | 0.182 | *r_s_* = 0.12 | 0.362 | *r_s_* = 0.18 | 0.174 | *r_s_* = 0.18 | 0.182 | *r_s_* = 0.22 | 0.104 |
| Δ Phenylalanine | *r_s_* = 0.07 | 0.630 | *r_s_* = 0.16 | 0.223 | *r_s_* = 0.19 | 0.149 | *r_s_* = 0.29 | **0.029** | *r_s_ = 0.33* | **0.011** |
| Δ Proline | *r_s_* = 0.11 | 0.420 | *r_p_* = 0.25 | 0.065 | *r_p_* = 0.34 | **0.009** | *r_p_* = 0.35 | **0.007** | *r_p_* = 0.41 | **0.002** |
| Δ Serine | *r_s_* = -0.24 | 0.074 | r_s_ = -0.10 | 0.448 | *r_s_* = -0.13 | 0.318 | *r_s_* = -0.15 | 0.253 | *r_s_* = -0.23 | **0.079** |
| Δ Taurine | *r_p_* = 0.26 | 0.052 | *r_s_* = -0.14 | 0.290 | *r_p_* = 0.26 | **0.048** | *r_s_* = 0.17 | 0.198 | *r_p_* = 0.25 | 0.065 |
| Δ Threonine | *r_s_* = -0.20 | 0.142 | *r_s_* = -0.11 | 0.429 | *r_s_* = -0.08 | 0.573 | *r_s_* = -0.16 | 0.242 | *r_s_* = -0.07 | 0.602 |
| Δ Tryptophan | *r_s_* = 0.23 | 0.084 | *r_p_* = 0.24 | 0.075 | *r_p_* = 0.21 | 0.114 | *r_p_* = 0.29 | **0.029** | *r_p_* = 0.37 | **0.005** |
| Δ Tyrosine | *r_s_* = 0.01 | 0.930 | *r_s_* = -0.09 | 0.488 | *r_p_* = 0.27 | **0.041** | *r_s_* = 0.11 | 0.405 | *r_s_* = 0.20 | 0.141 |
| Δ Valine | r_s_ = 0.15 | 0.269 | *r_s_* = 0.15 | 0.263 | *r_s_* = 0.24 | 0.075 | *r_s_* = 0.24 | 0.071 | *r_p_* = 0.22 | 0.102 |

## Supplementary Figure S1

Supplementary Figure S1 title: White blood cell count in thousand/µL by dietary group at baseline and after four and eight weeks of the dietary intervention, respectively.


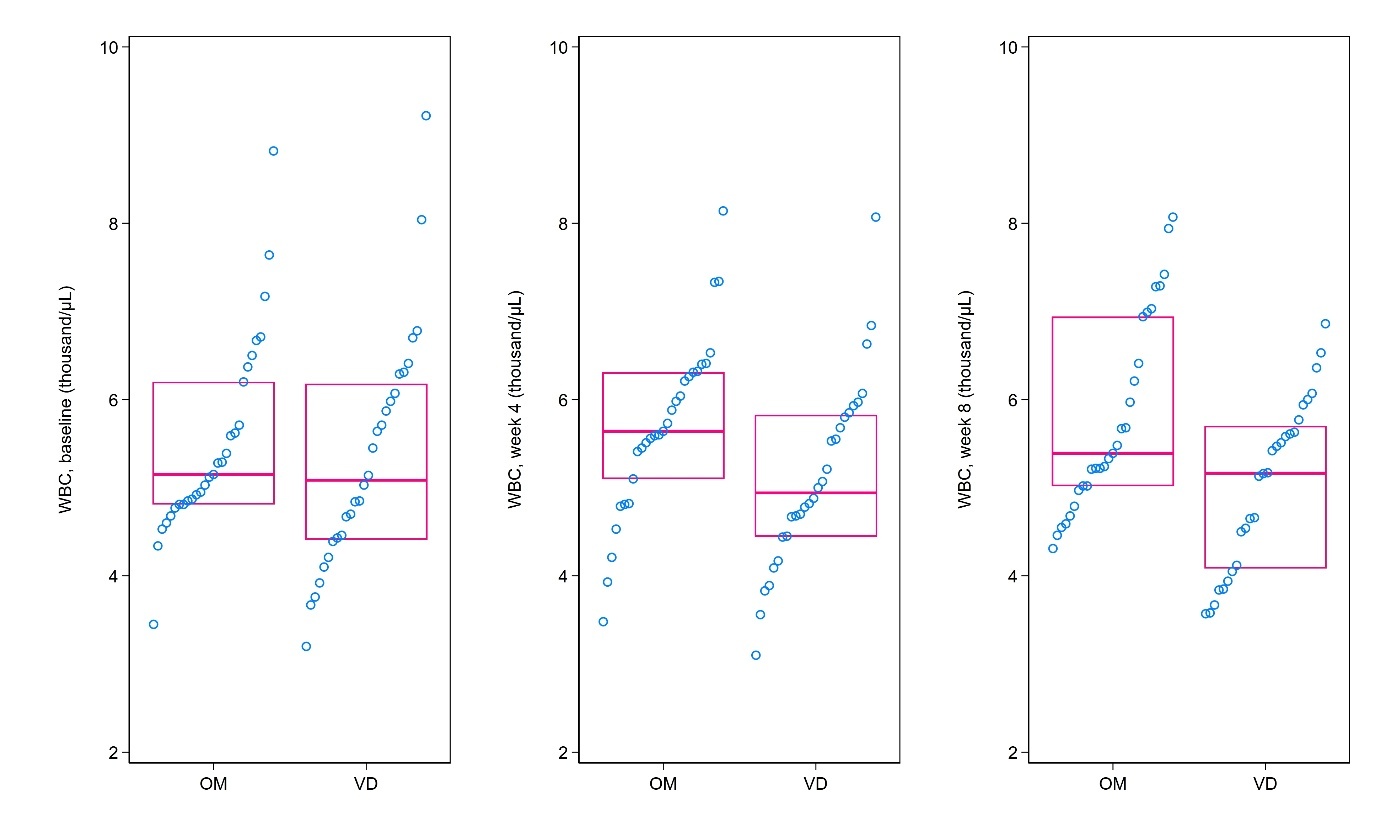


Abbreviations: VD: vegan diet, OM: meat-diet (omnivore diet enriched with meat, MD).

## Supplementary Figure S2

Supplementary Figure S2 title: Neutrophil count by dietary group at baseline and after four and eight weeks of the dietary intervention, respectively.


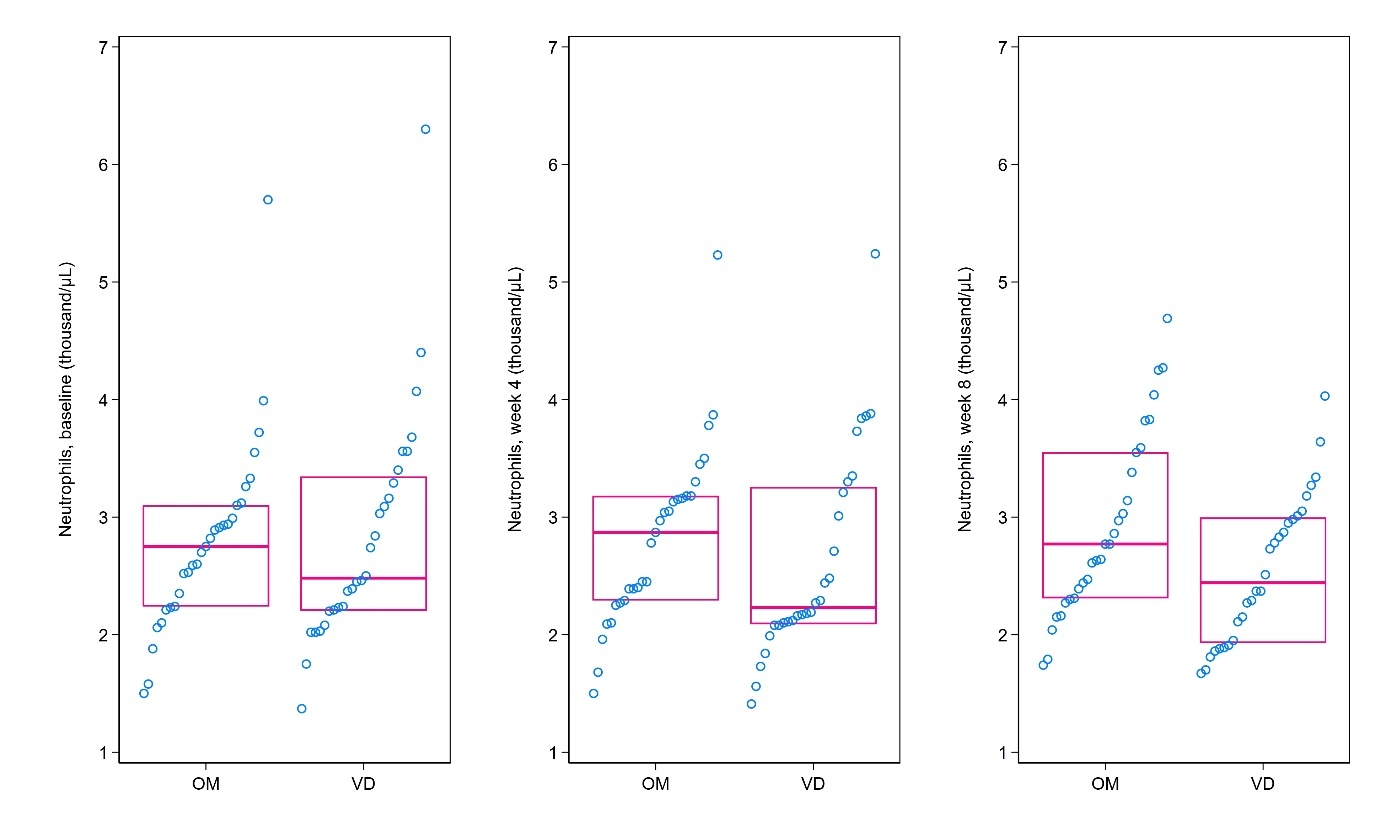


Abbreviations: VD: vegan diet, OM: meat-diet (omnivore diet enriched with meat, MD).

## Supplementary Figure S3

Supplementary Figure S3 title: High-sensitive C-Reactive Protein (hs-CRP) levels in mg/l by dietary group at baseline and after eight weeks of the dietary intervention.


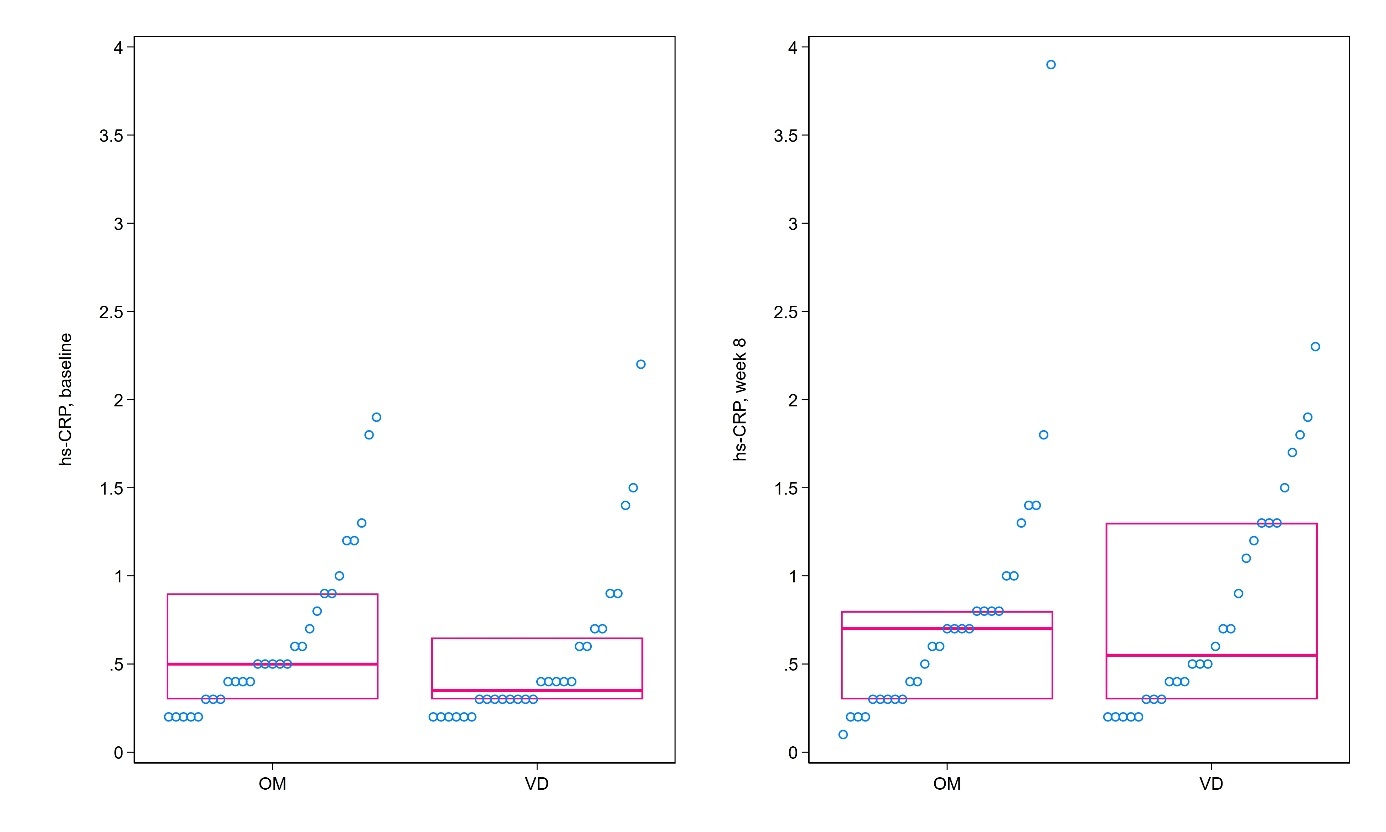


Abbreviations: VD: vegan diet, OM: meat-diet (omnivore diet enriched with meat, MD).

## Supplementary Figure S4

Supplementary Figure S4 title: Alpha-linoleic acid levels in µmol/l by dietary group at baseline and after eight weeks of the dietary intervention.


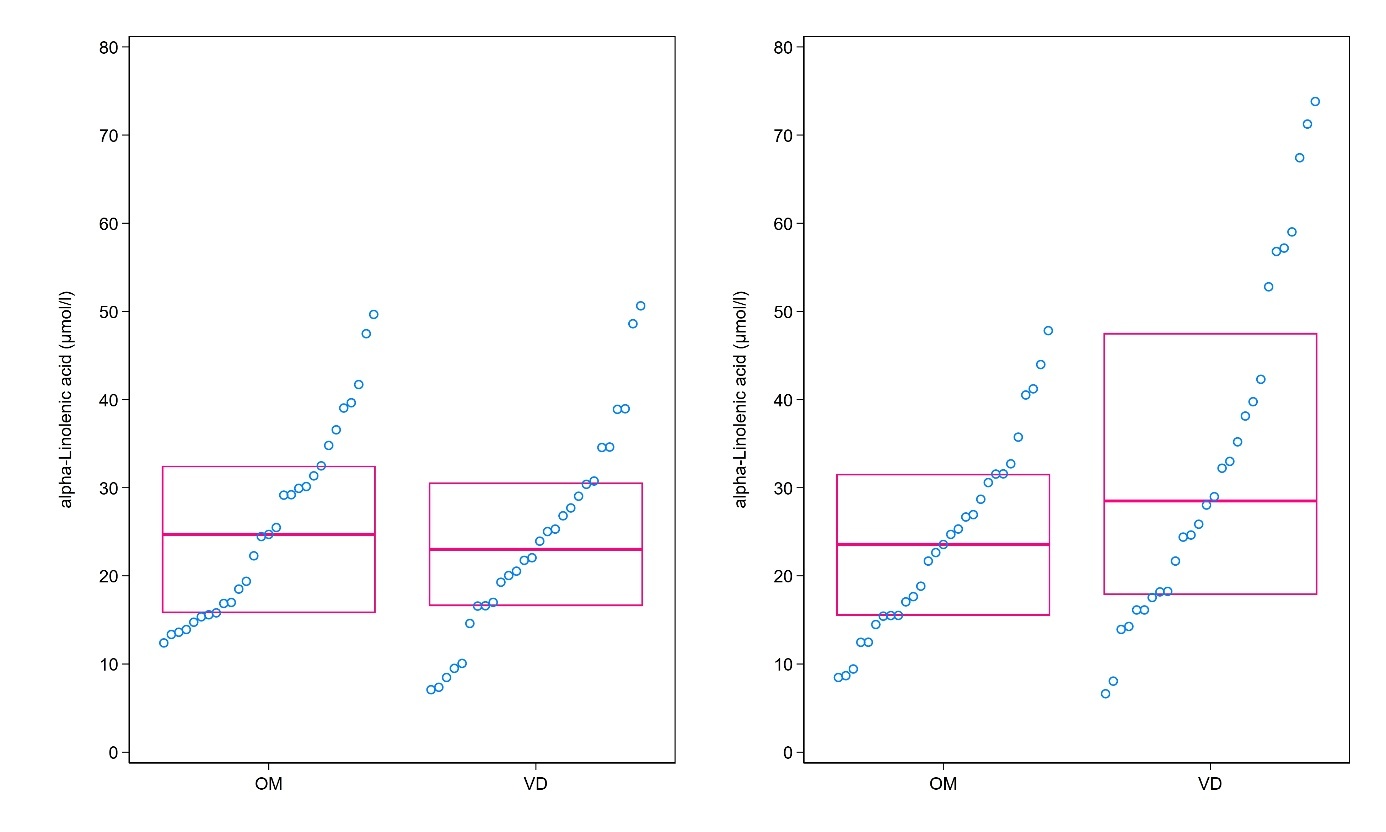


Abbreviations: VD: vegan diet, OM: meat-diet (omnivore diet enriched with meat, MD).

## Supplementary Figure S5

Supplementary Figure S5 title: 11-eicosenoic acid levels in µmol/l by dietary group at baseline and after eight weeks of the dietary intervention.


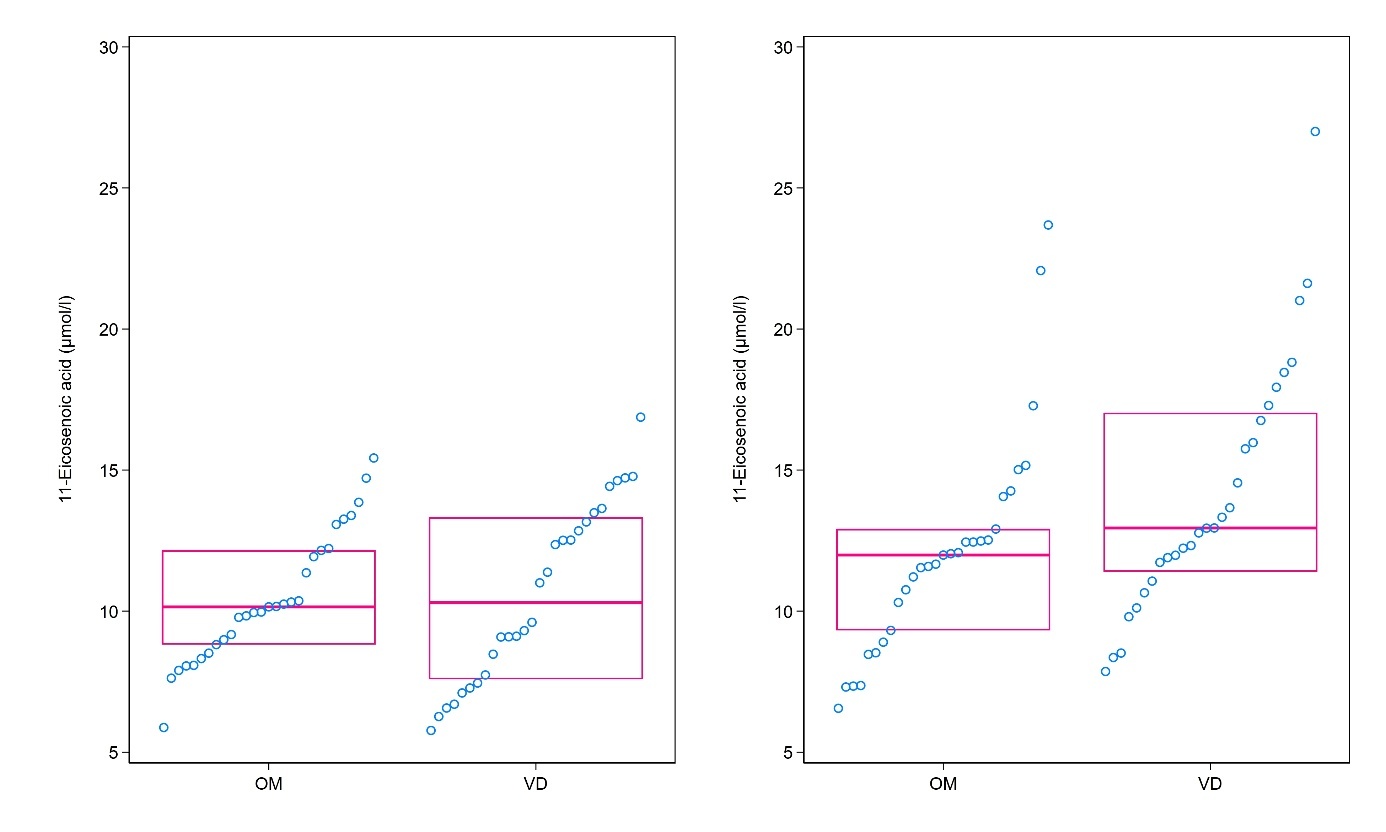


Abbreviations: VD: vegan diet, OM: meat-diet (omnivore diet enriched with meat, MD).

## Supplementary Figure S6

Supplementary Figure S6 title: Arachidonic acid levels in µmol/l by dietary group at baseline and after eight weeks of the dietary intervention.


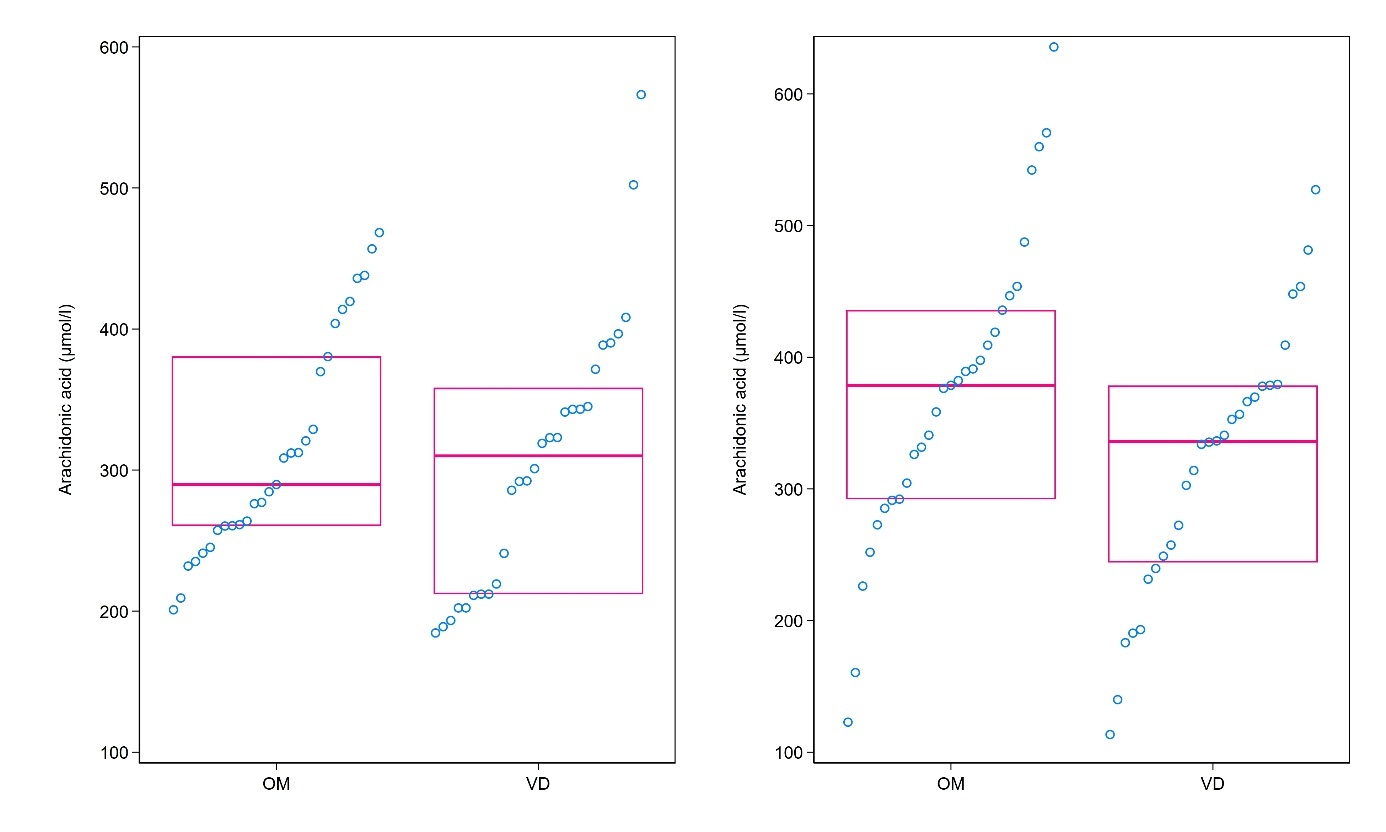


Abbreviations: VD: vegan diet, OM: meat-diet (omnivore diet enriched with meat, MD).

## Supplementary Figure S7

Supplementary Figure S7 title: Correlations between end-to-baseline differences of various amino acids (leucine/isoleucine, isoleucine 2, lysine and valine) and end-to-baseline difference in lymphocytes (panel A), white blood cells (panel B) and monocytes (panel C).


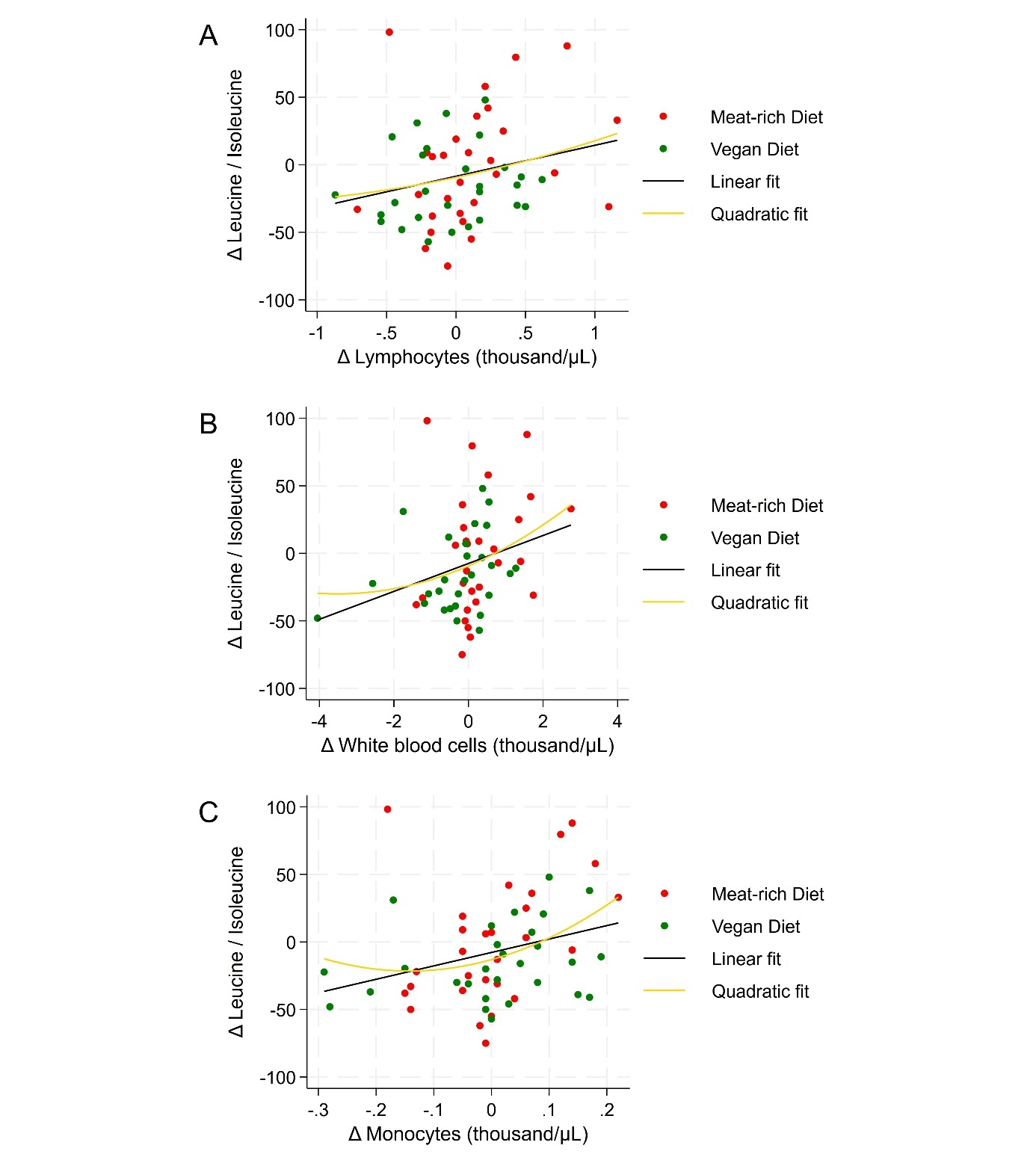


Positive associations between end-to-baseline differences of leucine/isoleucine and end-to-baseline differences of lymphocytes (r*_s_* = 0.27, *p* = 0.040), white blood cells (r*_s_* = 0.34, *p* = 0.010), and monocytes (r*_s_* = 0.35, *p* = 0.008) were found.
